# Understanding flux switching in metabolic networks through an analysis of synthetic lethals

**DOI:** 10.1038/s41540-024-00426-5

**Published:** 2024-09-17

**Authors:** Sowmya Manojna Narasimha, Tanisha Malpani, Omkar S. Mohite, J. Saketha Nath, Karthik Raman

**Affiliations:** 1 Centre for Integrative Biology and Systems mEdicine (IBSE), Indian Institute of Technology (IIT) Madras, Chennai, 600 036 India; 2 Department of Biotechnology, Bhupat Jyoti Mehta School of Biosciences, Indian Institute of Technology (IIT) Madras, Chennai, 600 036 India; 3 Department of Computer Science and Engineering, Indian Institute of Technology (IIT) Hyderabad, Hyderabad, 502 284 India; 4 Department of Data Science and AI, Wadhwani School of Data Science and AI (WSAI), Indian Institute of Technology (IIT) Madras, Chennai, 600 036 India; 5 Present Address: Neuroscience Graduate Program, University of California San Diego, San Diego, CA 92092 USA; 6 Present Address: Novo Nordisk Foundation Center for Biosustainability, Technical University of Denmark, 2800 Kgs. Lyngby, Denmark

**Keywords:** Metabolic engineering, Metabolic engineering

## Abstract

Biological systems are robust and redundant. The redundancy can manifest as alternative metabolic pathways. Synthetic double lethals are pairs of reactions that, when deleted simultaneously, abrogate cell growth. However, removing one reaction allows the rerouting of metabolites through alternative pathways. Little is known about these hidden linkages between pathways. Understanding them in the context of pathogens is useful for therapeutic innovations. We propose a constraint-based optimisation approach to identify inter-dependencies between metabolic pathways. It minimises rerouting between two reaction deletions, corresponding to a synthetic lethal pair, and outputs the set of reactions vital for metabolic rewiring, known as the synthetic lethal cluster. We depict the results for different pathogens and show that the reactions span across metabolic modules, illustrating the complexity of metabolism. Finally, we demonstrate how the two classes of synthetic lethals play a role in metabolic networks and influence the different properties of a synthetic lethal cluster.

## Introduction

Robustness in the face of environmental perturbations is an essential attribute of microorganisms^1–3^. This robustness is often achieved by the presence of multiple alternate pathways that achieve similar metabolic functions^4,5^. The redundancy introduced by alternate pathways comprises a large fraction of most metabolic networks^6–8^ and shows surprising variance in their distribution^9,10^. While some of the alternate pathways are very simple, arising due to gene duplication, other alternate pathways could be extremely complex, with compensating reactions spanning different metabolic subsystems^9,10^.

A straightforward method of studying these alternate pathways involves the identification of *synthetic lethals* in a metabolic model^10^. Synthetic lethals are sets of genes/reactions where only the simultaneous loss of all genes/reactions in the set leads to abrogation of cell growth^11^. When only one of the reactions is deleted, the cell is able to summon alternate pathways to ensure its survival. In many cases, this is made possible through a complex rerouting of fluxes in the metabolic network which exploits the redundancy in metabolism. However, very little is known about how these organisms reroute their fluxes, and how various reactions in the cell can compensate for one another.

Previous methods such as GIMME, iMAT, and RELATCH, for the study of flux distributions, have focused on a given condition, the final steady state of the cell, without considering the prior reference state of the organism^12–14^. REMI and deltaFBA are algorithms that integrate differential expression of transcriptome and metabolome with the flux distributions between two different states, a wild-type state and a mutant state^15,16^. These algorithms require gene expression data, which are not always available. Considering cells have a high order of redundancy and synthetic lethal, we require a method that can computationally predict the rewiring of metabolism. FBA alone has also been used^17,18^ to study redundancy using synthetic lethals in *E. coli* and other bacteria. In refs. ^17,18^, FBA was used to directly optimise the single deletions for the biomass objective and find the differences in the flux distributions. However, this can ignore the biological costs associated with altering flux in an organism, which may result in sub-optimal biomass production.

The resistance to antibiotics offered by the flexibility of metabolic rewiring has been shown to come with a fitness cost^19^. The advantages of alternate optimal solutions for various context-specific constraint-based modelling have also been illustrated before^20^. In anticipation of adapting to changing environments, bacteria opt for diauxic growth metabolism, which involves replicating at a sub-optimal growth rate so they can invest resources in new metabolic processes^21^. This sub-optimal growth is also seen in response to perturbations as the metabolic flux is rerouted in small adjustments first and then adaptively mutates to optimise for growth^22^. For the bacterium *B. subtilis*^23^, studies have shown that the relative change in flux distribution remains the same in mutant knock-outs even if absolute flux changes significantly. Thus, MOMA^24^, a sub-optimal growth rate-based FBA approach, uses a quadratic minimisation between the mutant and wild-type state to predict a sub-optimal growth rate.

FBA presents a challenge in the form of handling multiple flux solutions (even if there is a unique growth rate). To work around this and to identify this set of reactions that come into effect to rescue the cell from a non-lethal deletion, we propose a novel approach termed *minRerouting*. By solving a minimum *p*-norm problem, *minRerouting* can simultaneously solve for flux distributions that satisfy the stoichiometric constraints, maximise the biomass objective, and also minimise the number of reactions with varying metabolic flux values. We build on the wide body of evidence that supports flux balance-based predictions and further enhance the algorithm to account for the multiple solutions possible in any given conditions. The study further helps us understand the redundancies that facilitate robustness in wild-type organisms against perturbations and mutations. In addition, this approach is ideal for exploring and understanding indispensable changes in cellular metabolism between two different conditions, for instance, a healthy state and a diseased state, and has applications in the biotechnology and pharmaceutical industries.

Robustness arising from double lethal has been studied previously. It has been proposed that double lethal pair robustness in an organism can be ascribed to two classes of reaction pairs—plastic synthetic lethal (PSL) and redundant synthetic lethal (RSL)^18^. PSL pairs are reaction pairs where only one reaction is active, while the other reaction is inactive. The second reaction becomes active only when the first reaction is inactive. RSL pairs are reaction pairs where both the reactions are active simultaneously, yet, the loss of one does not abrogate growth. It has also been shown that these classes are conserved even across different nutrient conditions. The very presence of two distinct reaction pair classes calls for us to analyse the cause behind such selective activation. Are the inactive reactions more “metabolically costly” than the active ones? What kind of reactions make up the RSL pairs, especially when they are both simultaneously active? We employ a novel workflow to answer these questions and explore the structure of metabolic networks and their underlying redundancy. We also analyse the reaction types contributing to the PSL and RSL classes and uncover interesting patterns in their distribution.

## Results

The *minRerouting* pipeline has been carried out on eight genome-scale metabolic models. We have chosen organisms that represent key bacterial pathogens relevant to humans and are present in the BiGG database^25^. For *E. coli*l, found in the human gut, we have depicted two models, e_coli_core^26^ and *i*ML1515^27^. The model e_coli_core represents the simplified versions of only the most crucial pathways needed for its survival. The other models studied are for the bacteria *Helicobacter pylori, Klebsiella pneumoniae, Mycobacterium tuberculosis, Salmonella Typhimurium, Shigella sonnnei* and *Yersinia pestis*. These models and the number of single and double lethal reactions predicted for them using Fast-SL are listed in Table 1.

**Table 1:** List of all the models analysed as a part of the study

GSMM	Total number of reactions	Single lethals	Double lethals	Ref.
*Escherichia coli* (e_coli_core)	95	14	88	^26^
*Mycobacterium tuberculosis* (*i*EK1008)	1226	351	157	^68^
*Helicobacter pylori 26695* (*i*IT341)	554	252	54	^69^
*Escherichia coli* (*i*ML1515)	2712	253	287	^27^
*Yersinia pestis* (*i*PC815)	1961	212	189	^70^
*Shigella sonnei* (*i*SSON_1240)	2693	261	267	^71^
*Klebsiella pneumoniae* (*i*YL1228)	2262	199	144	^72^
*Salmonella enterica* (STM_v1_0)	2545	330	168	^73^

The third and fourth columns indicate the number of single lethals and double lethal pairs identified using Fast-SL^63,64^.

### Functional analysis of double lethals

#### Reaction submodules differ across organisms and within synthetic lethal pairs

Double lethals have been identified in many species previously, such as *E. coli*, *M. pneumoniae*, *S*. Typhimurium, and *S. sonnei* using FBA^17,18^. Our predictions are consistent with previous results regarding the number of synthetic double lethals obtained for different species and their composition of reactions. For *E. coli*, our results for synthetic lethals paralleled observations made by previous studies^17,18^.

Table 1 has the distribution of the number of single lethals and synthetic double lethals for each model. We see that the fraction of single lethal or essential reactions as compared to the total reactions is similar across models except for models of two species, *M. tuberculosis* and *H. pylori*, which have 29% and 45% of essential reactions. The number of double lethals also varies between the models, implying that the occurrence of a reaction as a double lethal is more nuanced.

**Fig. 1:**
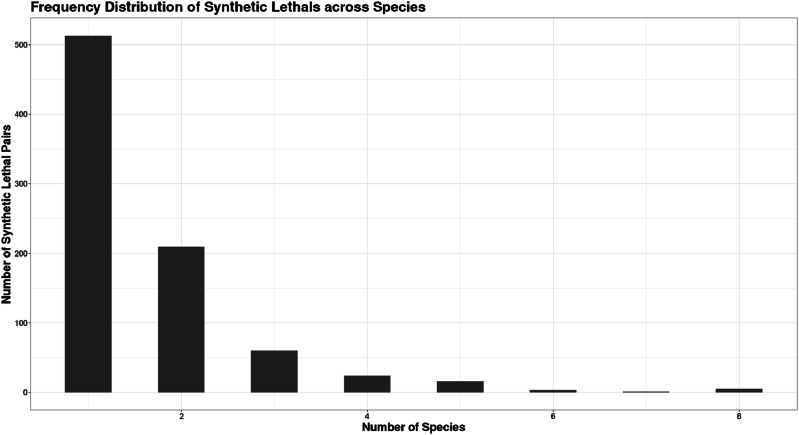
Distribution of common double lethal pairs across models. 500 reaction pairs are unique to specific models, while very few pairs are present across all eight models.

Figure 1 shows that more than 500 double lethal pairs are present in at least one organism, indicating the high level of redundancy present across metabolic networks. Among these, few are shared across at least five of the models analysed in the study. The tendency of a reaction pair to appear in multiple species with different adaptations implies that these could be potential super targets for drug therapies. The common synthetic lethal pairs examined across the organisms are all from the pentose phosphate pathway. The second most common reactions are from the glycolytic pathway and different amino acid biosynthesis pathways. Similar to observations made by Barve et al.^28^, the reactions that were essential belonged to linear or anabolic pathways such as ATP and histidine synthesis, while redundancies were present only in more reticulate pathways such as the pyruvate or glycolysis metabolic pathways.

**Fig. 2:**
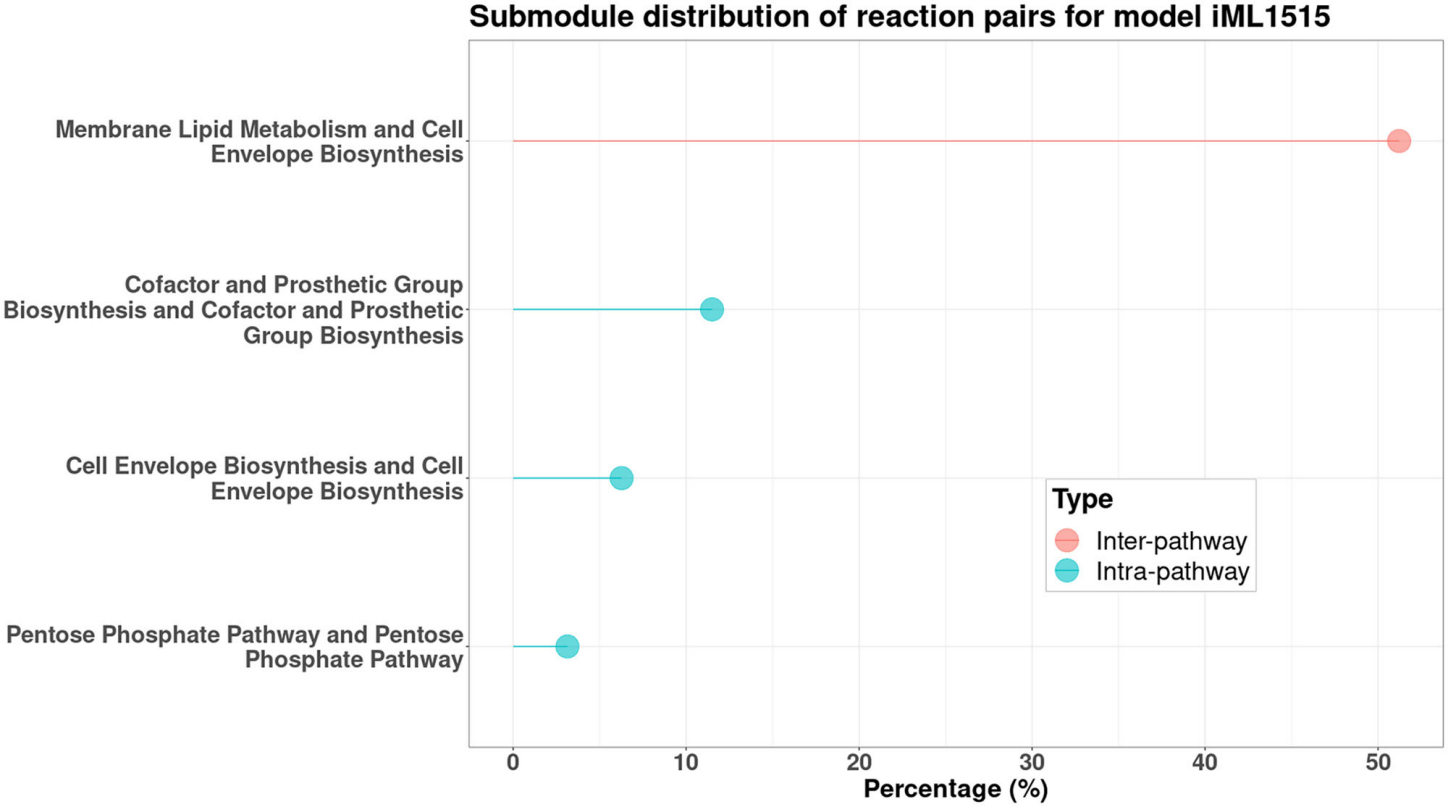
Metabolic subsystem analysis for the model *i*ML1515. The distribution shows that more than half the lethal pairs are from differing submodules. Only the submodules occurring more than the mean of the distribution are depicted for clarity.

**Fig. 3:**
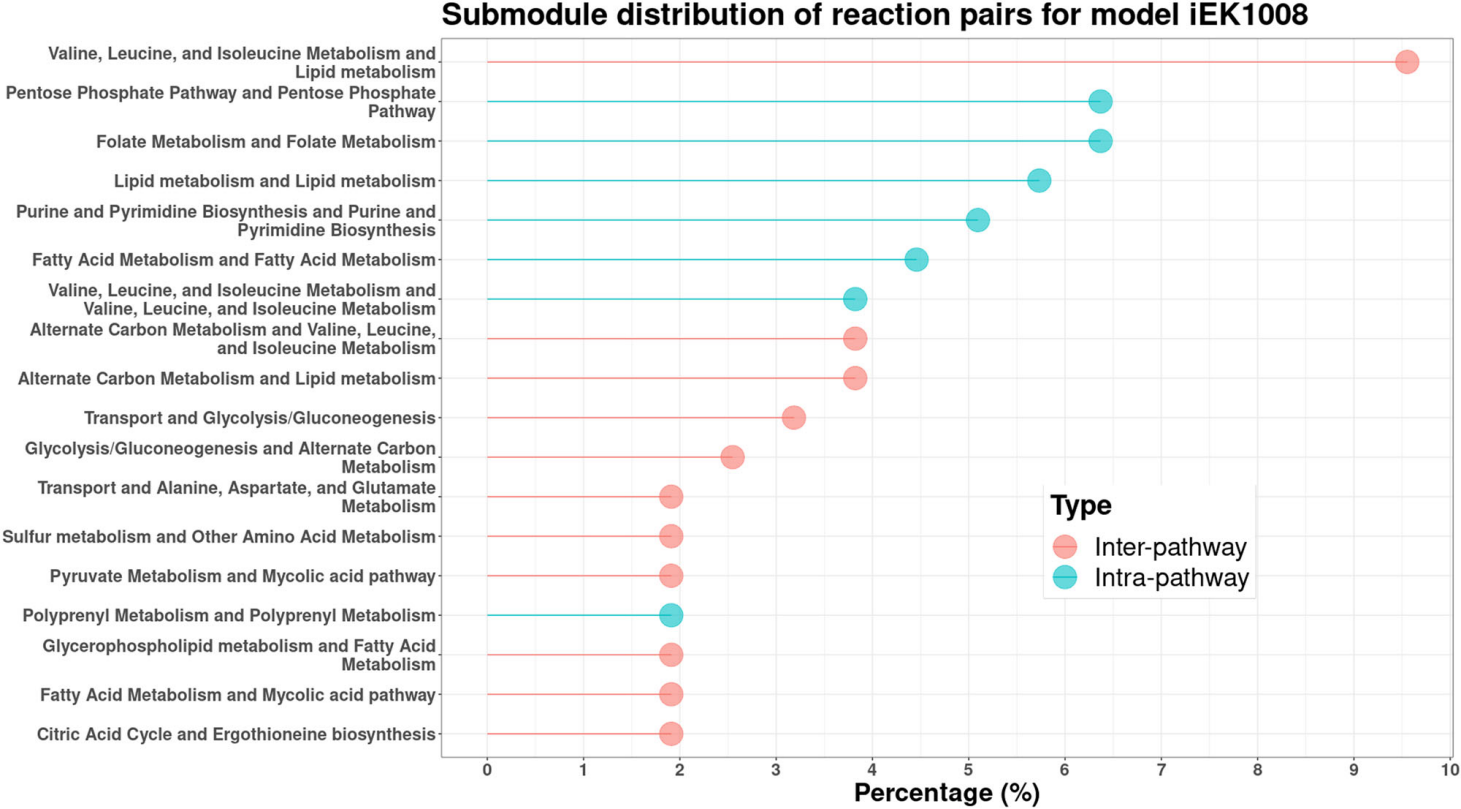
Metabolic subsystem analysis for the model *i*EK1008. The distribution shows lethal pairs from unique submodules, such as the mycolic acid pathway and pyruvate metabolism. Unlike *i*ML1515, cell envelope biosynthesis does not appear in any of the top pairs. Only the submodules occurring more than the mean of the distribution are depicted for clarity.

A broader understanding is obtained by looking at each organism’s submodule distribution of double lethal, as shown in Figs. 2 and 3 for the models *i*ML1515 and *i*EK1008. While it is intuitive to think the reaction pairs would arise from the same submodule, for all models, we see that at least 50% of the reactions are from different submodules, i.e., the synthetic lethals are inter-pathway. This could be because of the ripple effect caused by deleting one reaction, which causes small changes in all other connected pathways. Inter-pathway synthetic lethals also highlight an organism’s need for cross-talk between pathways, such as energy production and nucleotide metabolism^29^.

Previously, knock-out studies of reactions in the pentose phosphate pathway and glycolysis pathway have been performed. The flux rates of reactions for a *pyk* mutant in *E. coli* were quantified^30^ and summarised^24^. The results qualitatively matched the results of the PYK2-NDPK4 synthetic lethal pair’s flux distribution for the PYK2 mutant for 16 of 17 of the reactions in the *minRerouting* cluster set. The reaction that had a mismatch was the reaction PCK, which, even in wild-type *i*ML1515, was inactive. The above pathways have also been studied in strain-specific studies^31,32^, and the experiments show that reactions from the above pathways have similar redundancies as seen by *minRerouting*. Transaminases are another set of promiscuous enzymes catalysing the amino acid synthesis reactions. Their inter-dependency, which gives *E. coli* the ability to switch metabolic fluxes, has previously been studied^33–35^. These reactions for the *i*ML1515 model include VALTA, ALATA_L, VPAMTr, ASNS1, and ASNS2. These enzymes can perform underground metabolism and could be important biotechnological targets or drug targets. For the model *i*EK1008, two synthetic lethal pairs, namely, DHPS2-FOLD3 and TREP6PP-TREY, present in *M. tuberculosis* have been illustrated and shown in Supplementary Figs. 28 and 29.

In simpler and closely related bacterial systems, such as *E. coli* and *S. sonnei*, more than 50% of synthetic lethal reactions involve cell envelope biosynthesis, where membrane lipid metabolism reactions act as their backups. These systems also exhibit reaction pairs from the cofactor and prosthetic group biosynthesis submodule. *Y. pestis, K. pneumoniae*, and *S*. Typhimurium, phylogenetically different from the above two species^36^, additionally had reaction pairs from submodules associated with amino acid metabolism and glycerophospholipid metabolism. Finally, the specialist species *M. tuberculosis* and *H. pylori* have distinct dominant submodules, such as mycolic acid production and haem transport, respectively. The distributions for the other six models are given in the Supplementary Information. Thus, the redundancies are organism-specific, with double lethal reactions emerging from submodules required for biomass synthesis, directly or indirectly. Some submodules, like the pentose phosphate pathway, are shared in all organisms. These findings align with those from earlier experiments^10,17,18^.

#### Tendency of reactions in forming double lethal

**Fig. 4:**
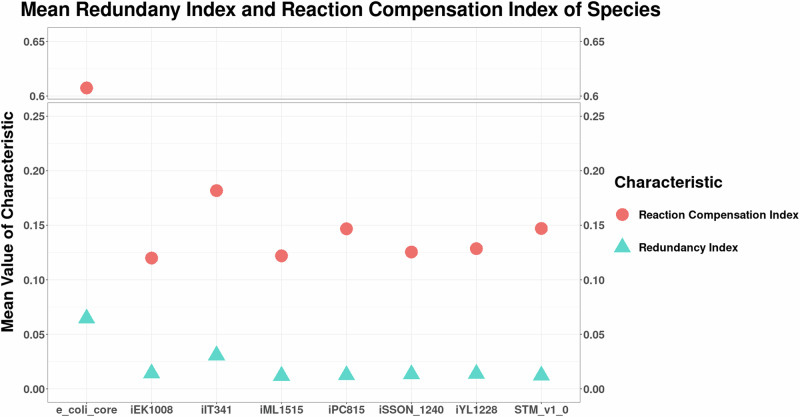
Mean redundancy index and reaction compensation index of reactions forming synthetic lethal pairs, across organisms. Reactions from e_coli_core have a higher RCI and RI compared to other models.

We next define the redundancy index (RI) for a reaction as the fraction of synthetic lethal pairs in which it occurs within a species (see subsection “Flux redistribution by synthetic lethals”). Several studies indicate that pathways lacking redundancy tend to have more critical functions for survival than those with redundant pathways^29,37^. However, there is evidence contradicting this notion^10,38^. Specifically, when a reaction is engaged in multiple pairs as a synthetic lethal and consequently possesses multiple backups, its function likely plays a significant role in ensuring the organism’s survival. Irrespective of the pathway, it has been shown that the essentiality of the metabolite determines the robustness of the related reactions, with the metabolite concentrations remaining unchanged to perturbations despite changes in incoming and outgoing reaction fluxes^39^. From Fig. 4, we observed that the models had a mean RI in similar ranges despite having different numbers of reactions and different adaptations. The reactions with a high RI were from different submodules for each organism, ranging from central carbon metabolism to transport and, more specifically, lipid metabolism for the bacterium *M. tuberculosis*.

A reaction is essential if it is necessary for growth in the given media^40^. Growth requires the production of bio-molecules, such as fatty acids, amino acids, and purine/ pyrimidine. The reactions that do not stop growth, or can be bypassed when eliminated turn out to be redundant. Thus, we saw that the reactions from submodules related to the production of the above bio-molecules occur in the single lethal list, and reactions that have redundancy in the form of double lethal were, albeit important, but from pathways that impacted biomass production indirectly. This concurs with the observations made about the reactions with a high RI. The *minRerouting* approach also reveals an essential characteristic of metabolic networks, i.e., the inter-dependencies of the pathways to produce biomass are revealed. These inter-dependencies are due to molecules with a high RI acting as connecting points for other pathways in a network. Suthers et al.^29^ investigated the topological structure of redundancy and observed comparable findings regarding the essentiality of various reaction modules.

Similarly, the tendency of a reaction to be part of a synthetic cluster, in the form of the fraction of synthetic clusters it occurs in, was calculated as mentioned in the subsection “Flux redistribution by synthetic lethals”. We have previously defined this as the reaction compensation index (RCI)^10^. The RCI shows similar trends as RI in terms of value, as seen in Fig. 4. However, the reactions that have a high RI do not necessarily have a high RCI. A high RCI indicates reactions that are important for all the key pathways that the synthetic clusters form, but these are not necessarily essential at least at the order of a double lethal.

To further understand how these reactions compensate for each other by rerouting flux, and ensure their viability, we further analysed the results from the *minRerouting* algorithm.

#### Effect of media/environment on single and double lethal

From previous results, we observed that flux rerouting occurs through diverse hidden pathways. To ascertain the influence of the environmental conditions on metabolic rewiring and associated costs, we changed the media concentrations in two ways. First, we changed the concentration of up to 10 carbon sources as described in the “Methods” section. Second, we looked at the effect of unblocking all exchange reactions in the model to varying degrees, i.e., −100, −500, and −1000 mmol g DW^−1^ h^−1^. In total, the results for four new models (Model_carbon, Model_exchange_1, Model_exchange_2, and Model_exchange_3) were compared to the original for all eight species and the results are given in the Supplementary Information. From Supplementary Table 3, we see that the number of single lethals decreased for all the models where exchange reactions were unblocked. The carbon sources had little to no effect on single lethals. This is intuitive since the single lethal reactions are directly linked to biomass production (amino acid synthesis and purine and pyrimidine metabolism). From Supplementary Table 4, we see that the effect of environment on double lethals was once again more varied for each species, and the number depended on whether the carbon source was present in the model and the number of exchange reactions in the model.

However, there is little overlap between the single and double lethal reactions between the different environments. As seen from Supplementary Fig. 22 for *E. coli*, the model where carbon sources are changed in the media has the most fraction of reactions in common with the original model. However, by adding exchange reactions to the media, the need for synthesis of amino acid and purine/ pyrimidine synthesis was removed. Hence, the submodules for single lethal in the new models consist of reactions mainly from the cofactor and prosthetic group biosynthesis or the lipopolysaccharide biosynthesis/ recycling submodules. Similarly, the need for central carbon metabolic pathways was removed, and we see from Supplementary Fig. 25 that the double lethals related to the pentose phosphate pathway and lipid metabolism disappeared. Instead, the transport reactions become redundant and form synthetic lethal pairs.

In *M. tuberculosis*, as seen in Supplementary Figs. 23 and 26, where the number of DLs increases, the trend remains the same with the decrease in dependency and, hence, a decrease in redundancy on the pentose phosphate pathway and the lipid metabolism pathways. A unique example of change in the double lethal is for *S. enterica* when the exchange reactions are switched on, and specifically, the reaction ENLIPAtex, present only in *S. enterica* is switched on. From Supplementary Fig. 27, we can see that this reaction enables the circulation of the metabolites from the Lipopolysaccharide Biosynthesis Recycling pathway. Thus, a major portion of the double lethal is from this pathway. In all the species, we see an increase in the double lethals related to transport pathways due to the increase in richness of the environment media. The Supplementary Information depicts the observations for three models for simplicity. The reader can repeat the analysis for the other models using the code available.

### Flux redistribution analysis

For each of the *p*-norms, the resultant *minRerouting* set is analysed. The properties described in the subsection “Flux redistribution by synthetic lethals” are studied in the following sections.

#### Stricter optimisation constraints expectedly yield smaller *minRerouting* sets

The size of the minimal rerouting set, as discussed in the “Introduction” section, is the number of reactions through which flux is rerouted. *minRerouting* allows the user to input their preferred norm to minimise the rerouted flux since differences in the calculation of the norms result in slightly different results. The *L*_2_ norm is called the least squares error norm as it minimises the sum of the squares of the differences. As a result, it tends to be influenced by outliers which can lead to unexpected solutions. However, its solution is unique and stable. On the other hand, the *L*_1_ norm gives a sparse solution, but with the possibility of multiple solutions for the minimisation problem. The *L*_0_ norm, computationally difficult to calculate, also results in a sparse solution. The comparison of the *minRerouting* cluster size across three norms, shown in Supplementary Fig. 4, thus reveals that the *strictest* *L*_0_ norm optimisation results in the smallest SL Cluster Size, followed by the *L*_1_ and *L*_2_ norms, respectively.

**Fig. 5:**
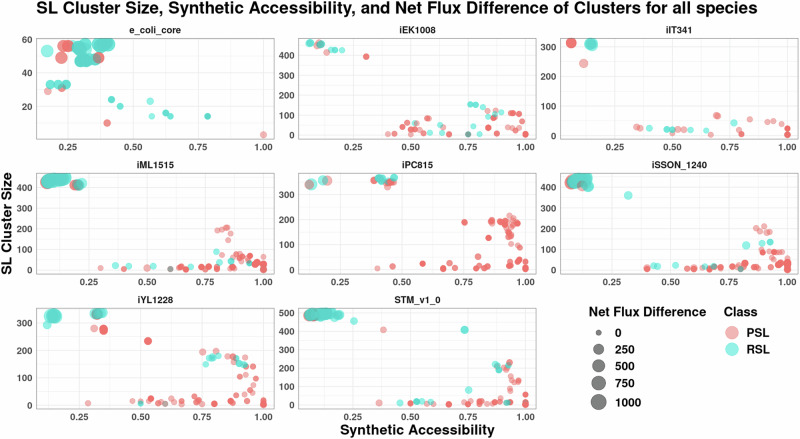
Cluster size, synthetic accessibility, and the net flux difference between the two metabolic states of reaction pairs. Mostly, the pairs that have a high synthetic accessibility have a low net flux difference and small cluster size. Outliers showing different properties exist in the top left corner for all the models except e_coli_core.

Interestingly, the sizes of the clusters calculated from *minRerouting* for *L*_1_ norm are lesser than observations made by Massucci et al.^17^ as seen in Supplementary Fig. 12. In stressful environments, restructuring metabolism incurs functional and structural costs. Our approach minimises the alterations in flux required, even if it means a partial reduction in growth rate, such that the expenses are reduced. Thus, a smaller cluster size is expected. The cluster size for each double lethal pair of an organism is shown in Fig. 5, along with other properties of the cluster for easy comparison across models.

#### Size of the common *minRerouting* Set indicates the efficiency of rerouting

The size of the common minimal rerouting set or synthetic lethal cluster, as discussed in the “Methods” section, is the number of common reactions through which flux is rerouted. The comparison of the common *minRerouting* Set size across norms is shown in Supplementary Fig. 4.

Once again, the results obtained show that the *L*_0_ norm optimisation results in the smallest Common SL Cluster Size, followed by the *L*_1_ and *L*_2_ norms, respectively. In certain models, the median Common SL Cluster Size is 0. This indicates that the *L*_0_ norm, in addition to ensuring minimum SL Cluster size, forces the two flux vectors to take completely exclusive reaction pathways, with no reaction (with modified flux) overlap. Since the organism undergoes complete rewiring, the number of distinct reactions flux is rerouted through between the two flux vectors, normalised over the total number of reactions undergoing a flux change has been termed *Synthetic Accessibility*. The organism has to divert its energy into activating/deactivating the reactions not common between the two reaction pair deletions. A Synthetic Accessibility of 1 would imply no common reactions between the metabolic states of a synthetic lethal pair. While the range of Synthetic Accessibility is vast, on average, at least 25% of reactions in a cluster are turned on/off when switching states. A low Synthetic Accessibility could mean that the function of the reaction is not being completely replaced but is being compensated for by a collective change in a group of reactions mediated through its pair. A high Synthetic Accessibility could mean that most of the reactions that get activated in the mutant are not needed in the wild-type state.

In literature, there are multiple schools of thought on the role of redundancy in metabolic networks. True redundancy is believed to not be possible as it is evolutionarily unstable^41,42^. A genuinely redundant gene coding for the redundant reaction would be lost completely through genetic drift since its mutation would incur no fitness costs to the organism. Hence, we see that synthetic accessibility indeed has a vast range. However, the clusters with a small cluster size tend to have a high Synthetic Accessibility. Thus, the backing is efficient, and reactions are not unnecessarily active. However, one group of clusters is unique, as seen in the top left corner of every model in Fig. 5. They are outliers that have a high cluster size and low synthetic accessibility. Thus, Synthetic Accessibility gives us an idea of the efficiency of the reaction pair in the metabolic network and the cost of replacing that function that the organism is willing to bear, even with a decrease in its growth rate.

#### Net flux difference is indicative of the extent of rerouting

The flux difference is the total flux difference between the two flux vectors representing the deletion of individual reactions that make up the double lethal. The net flux difference across norms is compared in Supplementary Fig. 5. Flux difference shows the same trend for *L*_0_, *L*_1_, and *L*_2_ norms. As seen in Supplementary Fig. 12, the net flux difference between reaction pairs obtained using our *L*_1_ norm formulation is significantly lesser than that reported by Massucci et al^17^. The flux difference gives an indication of the cost of maintaining the redundancy between the reaction pairs. *minRerouting* thus reveals the flux redistribution that the organisms can undertake to decrease this cost further. Since the net flux difference is comparable between the norms while the cluster size is more for *L*_2_, the average change in a reaction flux is lower for *L*_2_ norm than *L*_1_ and *L*_0_ norm. This may be because the *L*_2_ norm penalises differences more as it squares them to obtain the error.

The average flux difference of clusters is high in the e_coli_core model, due to its limited ability to adjust to perturbations. Thus, even though organisms do not need many reactions to exist, more reactions provide robustness to the network.

For each pair of a subset of synthetic lethals, we also generated the corresponding knockout models—modelDel1 and modelDel2. From the flux space generated by flux sampling of these two metabolic states (2000 flux distributions each) using Flux Sampling Analysis (FSA)^43^, we identified the flux distributions between the two that produced the minimal change in the p-norm for *L*_0_, *L*_1_, and *L*_2_-norms. This difference, generated from the flux sampling space, was compared to our observed flux difference values generated from minRerouting. As can be seen from Supplementary Figs. 30 and 31, flux sampling fails to perform as well as minRerouting in terms of minimising the change between two mutant states but does better for *i*IT341 than for *i*EK1008. This is probably because we only took 2000 flux solutions while *i*EK1008 has more reactions than *i*IT341 norm, and thus, a sample size of 2000 captures its solution space better.

**Fig. 6:**
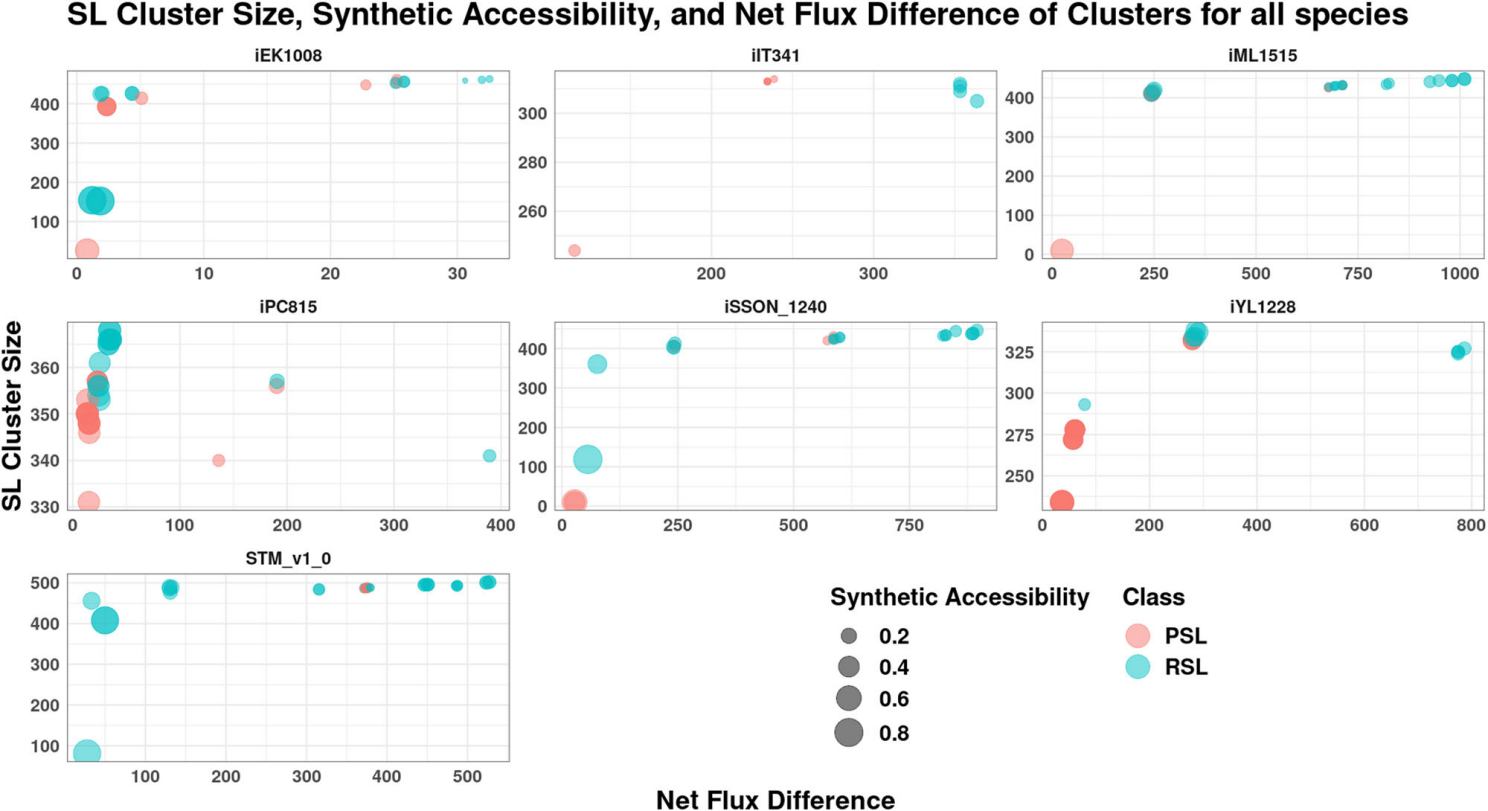
Cluster size, synthetic accessibility and the net flux difference between the two metabolic states of outliers across species. Here, we can see that RSLs have the highest flux difference owing to their cluster size. Pairs with higher synthetic accessibility have lesser flux differences despite large cluster sizes.

The outlier groups for all models barring e_coli_core, mentioned previously as the top left bubbles, consisting of clusters with a low Synthetic Accessibility and high cluster size, also have a high net flux difference which can be seen in Fig. 6. From the bubble plot, we see that the outliers exist as two subgroups in each species. The outliers that still have a lesser flux difference all have higher Synthetic Accessibility and lesser cluster size, as seen in the bottom left of each panel. The ones with higher flux difference are the second group with lower Synthetic Accessibility and bigger cluster size, as seen in the top right corner of each panel. The pentose phosphate pathway module and reactions from various transport submodules show up as outliers in many of the organisms, along with species-specific reactions. In *K. pneumoniae*, we see histidine metabolism as a replacement for the TCA cycle as a major subgroup of the outliers. For *M. tuberculosis* we see the reaction space from mycolic acid metabolism, and for *H. pylori*, reactions from the urea metabolism are present in the outliers. These outliers are important reactions of interest since they are not bypassed even though their deletion causes a major disruption in the flux distribution of the organism.

#### Effect of media/environment on flux rerouting in synthetic lethals

The characteristics of minRerouting studied for the models also change when the media concentrations change. As seen in Supplementary Fig. 22, due to a change in environment that led to more reactions being activated, the cluster size almost always increased without an increase in synthetic accessibility. This may indicate that organisms prefer rerouting flux through already active reactions instead of switching on a new reaction. This preference (small synthetic accessibility) may have to do with the need for impromptu transcription and translation of the mRNA for the new reaction. These results are consistent across the models. On the other hand, though minimised, the flux difference shows extremely large values mainly due to a general increase in the flux rates of reactions, including the growth rate. Thus, the difference in flux between alternate routes carrying a high flux will result in a high flux difference.

The rerouting also occurs with two strategies: PSLs and RSLs. What difference does the plasticity or redundancy make to the observations we have seen? We next check how they impact the robustness of metabolism.

### Metabolic efficiency analysis

We next analysed the metabolic efficiency of the reactions that comprise double lethal pairs, based on pFBA reaction types^40^. The relation between these types and the class of synthetic lethal pair has also been studied and the results obtained have been elucidated below.

#### High occurrence of PSLs over RSLs

**Fig. 7:**
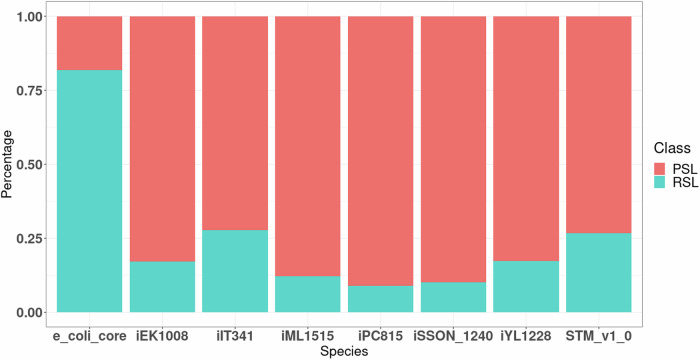
PSL-RSL distribution across models. While in most models, the fraction of PSLs is much greater than that of RSLs, the trend is reversed in the case of the model e_coli_core. This could be expected because e_coli_core only comprises the metabolic core of *E. coli*, and hence, most of the double lethal pairs comprise reactions that require to be simultaneously active.

Using flux variability analysis and the methodology described in the subsection “Plasticity and redundancy in synthetic lethals”, we have classified the clusters into redundant synthetic lethals (RSLs) and plastic synthetic lethals (PSLs). This classification of synthetic pairs is based on the two strategies taken by the synthetic pairs to maintain robustness. It helps explain different properties seen till now concerning cluster size, flux difference, and composition. Previous analysis^17,18^ has revealed that PSLs are the more complicated way of acquiring redundancy, needing sophisticated functional organisation with fewer resources, while RSLs represent a more rudimentary strategy. For simple species like *E. coli*, their results showed that RSLs had more intra-pathway submodule pairs, PSLs had more inter-pathway submodule pairs, and for *M. pneumoniae*, no pattern was observed for RSLs or PSLs. From Fig. 7, we see that the number of RSLs is higher than PSLs only in the e_coli_core model, which is the simplified metabolic network of *E. coli* without any functional or structural complexities of metabolic networks. Thus, organisms tend to promote the plasticity of networks. The clusters do not prefer inter-pathway or intra-pathway reaction pairs but seem to depend more on the organism and which pathways are important to it. In *E. coli*, the most common submodules for pairs were inter-pathway due to their dependency on the cell envelope synthesis pathway and the membrane lipid biosynthesis pathway.

From Supplementary Fig. 13, we see that PSLs exhibit a smaller cluster size for each species than RSLs. This smaller cluster size indicates that transitioning between two states is structurally simpler, with fewer changes needed. This ease of transition is also reflected in the functional costs, as PSLs have a lower flux difference between the two states, as seen in Supplementary Fig. 15. The difference in costs and efficiency is highlighted by the higher Synthetic Accessibility of PSLs as compared to RSLs in Supplementary Fig. 14. Again, these trends are not seen in e_coli_core, which is unique due to limited flexibility stemming from its lesser reactions and connectivity.

For *E. coli*, we compared the expression of RSL and PSL genes across contexts for 10 synthetic lethal pairs each, as seen in the iModulonDB^44^. The gene co-expression patterns are represented as heatmaps in Supplementary Figs. 32 and 33. For the subset of lethals analysed, RSL genes (given in Supplementary Table 2) were co-expressed, though not always to the same extent. Specifically, genes coding for the reactions RPI (*b2914, b4090*), RPE (*b3386*), TKT1 (*b2935, b2465*), and TKT2 (*b2935, b2465*) co-expressed together as these form synthetic lethals with each other. Conversely, PSL genes (Supplementary Table 1) exhibited differential expression for at least two genes coding for the two reactions in a pair. From the heatmap, we can also see that some reactions, such as AACPS3, were expressed in a few environmental conditions, and the corresponding lethal pair reaction 3HAD160 was expressed in mutually exclusive other conditions. Thus, the classification of synthetic lethal as RSLs or PSLs may be dependent on environmental conditions as well.

While the RSLs and PSLs help us understand the above trends, why does the network have differing strategies? Why do not the RSL reactions in a pair compete against each other, with only one reaction becoming evolutionarily stable? In PSLs, why does the inactive reaction stay in the network when it is not needed in wild-type scenarios? To understand why this happens and dive deeper into the types of reactions that contribute to RSL and PSL pairs, the metabolic efficiency analysis of the reactions was performed using the reaction classes returned by pFBA^40^.

#### PSLs and RSLs constitute distinct classes of reactions

**Fig. 8:**
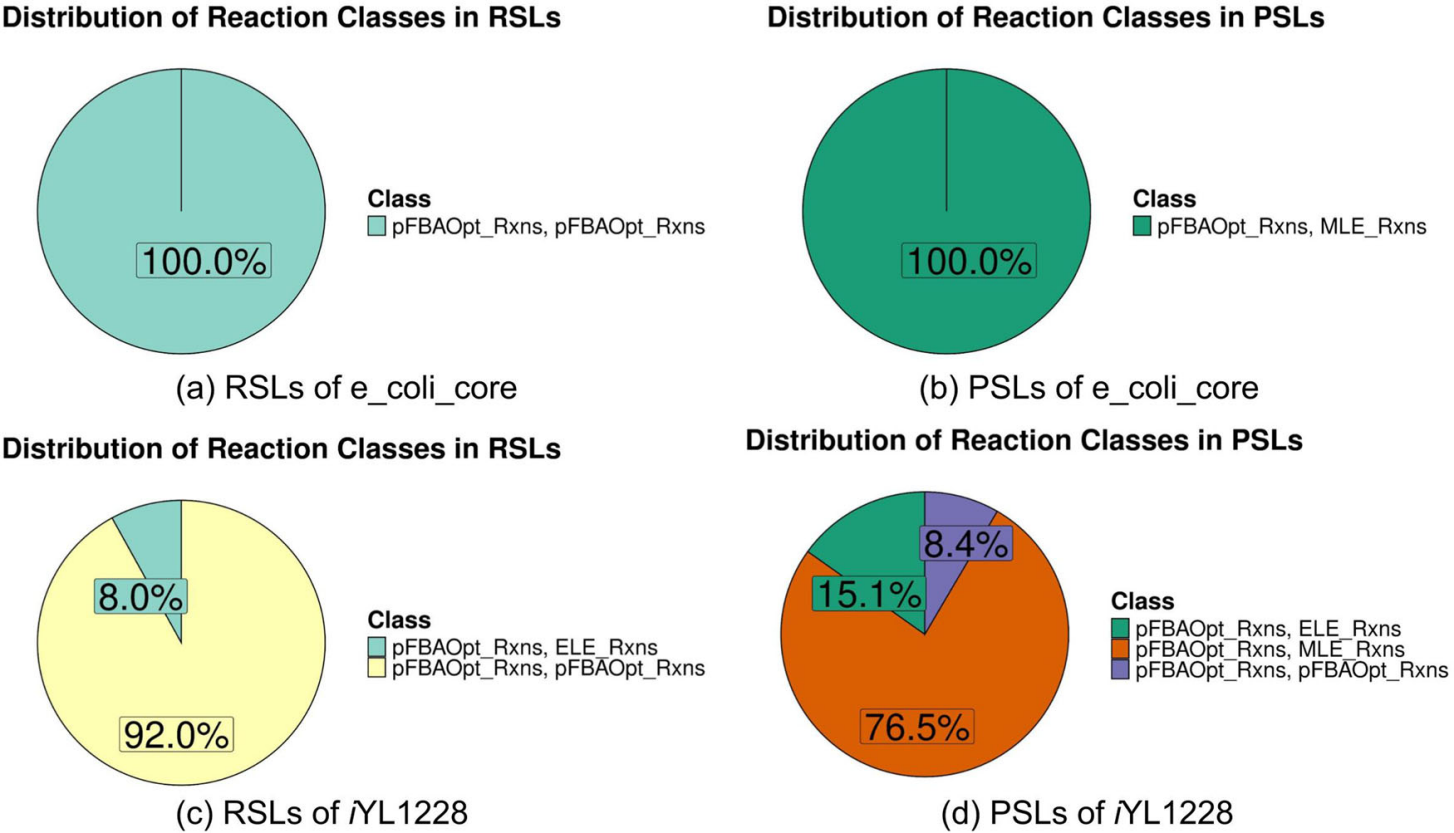
Schematic of reaction pair distribution between the RSL and PSL classes for e_coli_core and *i*YL1228. **a**, **c** Majority of the reaction pairs that are classified as RSL pairs are (pFBA optimal, pFBA optimal) pairs. **b**, **d** At least one reaction is pFBA optimal in PSLs. The distribution for the rest of the models can be accessed in the supplementary results provided in the Supplementary Information.

Using the approach from subsection “Parsimonious FBA (pFBA)”, we see how the type of reaction, pFBA Optimal, blocked, enzymatically less efficient (ELE), metabolically less efficient (MLE), zero flux reaction, or essential reactions, influenced double lethal pair formation. From Supplementary Fig. 2, we see that the Redundancy Index does not depend on the type of reaction. In fact, we see a high composition of ELE and MLE reactions to be redundant which is unexpected yet concurrent with the study by Wang et al.^38^. We also see that the percentage composition of RSL pairs is lower than PSLs, possibly because of the more evolved nature of PSLs. Yet, the RSLs are found to be composed of mainly pFBA Optima reaction pairs (Fig. 8). In PSLs, while one of the reactions was pFBA Optima (possibly the active reaction in wild type state), the second reaction was seen to be part of any of the other types of reactions. The reason reactions other than pFBA Optima form redundant pairs in nature is puzzling but can be explained by various evolutionary forces acting simultaneously over the organism while under the influence of its environment^38^. For *E. coli*, we found the same PSLs to repeat in constraint-based computational experiments^9^. We found one PSL pair to have been experimentally validated—ASNS1 and ASNS2, with the former being the efficient active enzyme under normal growth conditions^45^. In literature, we found two enzyme studies that described the efficiency of PSL pairs in *M. tuberculosis*. One of these pairs is depicted in Supplementary Fig. 28. Previous studies show that DHPS2 is the main enzyme catalysing the formation of folate, an important co-factor, but FOLD3 is present as a less efficient enzyme for the same function^46,47^. Similarly, there exist two thymidylate synthases in *M. tuberculosis* that catalyse the biosynthesis of deoxythymidine monophosphate (dTMP or thymidylate). The product of *ThyA* (TMDS) is the efficient enzyme that is backed up by *ThyX* (TMDS3)^48,49^. The role of these redundant processes in the resistance to the drug *para*-aminosalicylic acid highlights the need for *minRerouting* to predict alternate pathways of drug targets that may lead to drug resistance^50^.

From the metabolic efficiency analysis, it is clear that the type of reaction influences the formation of different classes of synthetic lethals and, in turn, its properties. Comparisons between e_coli_core and *i*ML1515 also reveal that the extent of connections and density of the network also impact the rerouting of metabolism. The usage of our algorithm and these results indicate how the robustness of metabolic networks is more complex than anticipated.

## Discussion

In this study, we have looked at how organisms, especially pathogenic bacteria, rely on the presence of redundancy in the form of synthetic lethals to make themselves robust against genetic perturbations. With the help of synthetic lethals and our proposed algorithm, *minRerouting*, we have analysed how flux is redirected in metabolic networks upon perturbations. Mahadevan and Lovley^4^ had previously established a relationship between the mode of metabolism and their redundancy. Typically, specialists do not rely on alternative pathways as compared to generalist species. From our results, we see that the bacteria have differing numbers of single and double lethals, implying differing strategies for ensuring robustness. However, more analysis needs to be carried out to further strengthen this hypothesis. These synthetic lethals differ in functionality as well. Yet, there is a set of synthetic lethals that are common across species. These are related to the central carbon metabolism, like the pentose phosphate pathway. Reactions that are from these common pathways are important as their functionalities could be potential targets for drug therapy and combating antimicrobial resistance. They represent nodes in the metabolic network that interact with multiple other nodes simultaneously.

Our optimisation formulation is used to obtain the minimal set of reactions through which fluxes are rerouted. We used three different approaches based on three norms—sparse (*L*_0_), linear (*L*_1_) and quadratic (*L*_2_) approaches—and obtained different *minRerouting* sets for each of the norms. It is likely that the *L*_0_ captures the final steady state of the cell post-adaptation to the knock-out, while the other norms capture the transient response of the cell to the perturbation, i.e., gene deletion, similar to results observed by Shlomi et al.^51^.

We also showed that the size of the *minRerouting* set is the smallest when the sparse formulation is used and learned that sparsity forces the use of exclusive reaction rerouting, which results in a null common rerouted reaction set (see Supplementary Fig. 4).

We next proposed a systematic, conditional FVA approach to classify double lethal pairs into PSL (Back-Up Reactions) or RSL (Parallel-Use Reactions) reaction pairs. Parallel to remarks made previously^18^, the Synthetic Accessibility of PSLs highlights their sophisticated nature in contrast to RSLs, and in all models barring e_coli_core, the PSLs were greater in number. These RSL and PSL pairs have differing properties in terms of the number of reactions through which flux is rerouted, the new set of reactions that become active when the flux is rerouted, as well as the costs of rerouting. We explored the reasons for such a disparity and hypothesised that the RSL pairs should have higher metabolic efficiency as both the reactions are simultaneously active, while reactions in the PSL pairs would have lower metabolic efficiency. The results of our study have proved that this was indeed true. The RSL reaction pairs of most of the organisms (excluding *Shigella sonnei*), comprise completely (or majorly) of (*pFBAOptimal*, *pFBAOptimal*) reaction pairs. As *pFBAOptimal* reactions are considered crucial for the growth of an organism, our hypothesis was validated. Reactions comprising PSLs must have come about by various evolutionary processes such as horizontal gene transfer, and pleiotropy and may not have been a result of being a back-up for adaptation or robustness. These observations were made possible by the results from the *minRerouting* algorithm.

An extension of *minRerouting* can be used to understand the complex metabolic reroutings that occur in several diseases. Particularly, in the case of cancer where the cells re-programme their metabolic activities, rerouting fluxes in such a way that they can continue to proliferate and maintain their malignant properties, *minRerouting* can help us understand these reroutings and perhaps help in finding better therapeutic cures. Potential synergistic effects of drugs can be unearthed from applications of this algorithm. For some cancers, synthetic lethal drug targets have been tested experimentally and computationally^52^. *minRerouting* has previously been used to identify how metabolic flux changes between two synthetic lethal pathways^53^. Appropriately, bacterial studies of synthetic lethals are also of importance. A previous study used constrain-based modelling to predict the drug targets of multiple *M. tuberculosis* drugs^54^. Some of these targets (*inha* and *fas*) are redundant, as seen by our analysis, and thus, this may lead to antibiotic resistance of *M. tuberculosis*. Gene expression experiments for drug treatment of *M. tuberculosis* with Isoniazid reveal many genes to be induced in response to the subsequent inactivation of the fatty acid synthases caused by the drug^55–57^. Isoniazid induces a shift from the TCA cycle to the fatty acid metabolism and mycolic acid synthesis. The increase in gene products of *cmaA2, mmaA2, mmaA3, mmaA4* (MYC1M1, MYC1M2, MYC1CYC4, and MYC1CYC5, MYC2CYC1, MYC2CYC2, MYC2CYC3) and *fbpC* (FBPA) seen experimentally match the overall reactions seen in the synthetic lethal clusters or rerouting sets of fatty acid synthase, FAS160, synthetic lethal pairs^55,57^. *Rc3140* (FADE233), and *ICL* (ICL), genes playing a role in the shift in the pathways above, are also seen in most of the synthetic lethal clusters in the *i*EK1008 model and have a high RCI of 0.35 and 0.43, respectively^56,57^.

The submodule distribution and the metabolic efficiency results take us one step closer to exploring the world of synthetic lethals present across metabolic networks. They reveal hidden dependencies between reactions and the influence they have on flux rerouting in a network. This approach of interpreting flux switching is crucial to understanding redundancies in metabolic networks and their differing roles.

## Methods

### Flux balance analysis

Flux balance analysis (FBA)^58,59^ is a constraint-based approach that is used to predict the steady-state flux distribution in a given organism’s metabolic network. FBA employs a linear programming (LP) formulation, with an objective to maximise the biomass flux under certain flux and stoichiometric constraints. The formulation of FBA is as follows:

1 $$\max \,{{\bf{c}}}^{\top }{\bf{v}}\,\,\equiv \,\,\max \,{{\bf{v}}}_{{\rm {bio}}}$$

2 $${{s.t.}}\,\,{\bf{S}}{\bf{v}}=0;\,\,{{\bf{v}}}_{{\rm {LB}}}\preccurlyeq {\bf{v}}\preccurlyeq {{\bf{v}}}_{{\rm {UB}}};$$

Here, **v** represents the flux vector, the *j*th entry corresponds to the flux through the *j*th reaction, and *c* represents the objective function. Typically *c*^T^*v* = **v**_bio_, where **v**_bio_ is the biomass flux, *S* represents the stoichiometric matrix of dimensions *m* × *r*, with *m* being the number of metabolites and *r* being the number of reactions, and **v**_LB_ and **v**_UB_ represent the permissible lower and upper bounds of the reaction fluxes. FBA has been experimentally validated in many scenarios^60,61^, and has widespread applications^62^. An important extension of FBA is the Minimisation of Metabolic Adjustment (MoMA)^24^, which seeks to identify a minimally different flux from the wild-type flux (by minimising the *L*_2_ norm of this difference). In scenarios where a reaction flux has been altered due to perturbations or additional constraints, MoMA finds the minimal changed flux values compared to the wild-type.

### Identification of synthetic lethals

Fast-SL^63,64^ is an efficient algorithm that identifies synthetic lethals by systematic pruning of the search space and exhaustive enumeration from the remaining reactions. Fast-SL rapidly identifies synthetic lethals and scales well for higher-order lethals. In this paper, we used Fast-SL to identify synthetic lethal reaction pairs in a given genome-scale metabolic model.

### minRerouting formulation

**Fig. 9:**
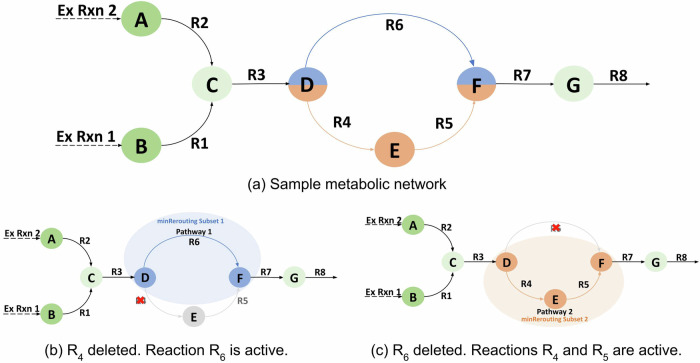
A sample metabolic network used to illustrate the concept of *minRerouting.* The nodes and edges are depicted as metabolites and reactions, respectively. This metabolic network comprises 10 reactions, 7 metabolites, and 3 double lethal pairs. The *minRerouting* Sets 1 and 2 are highlighted using orange and blue rectangular boxes, and the common reaction between the rerouting sets is *R*3.

Figure 9 represents a toy metabolic network comprising ten reactions and seven metabolites. The metabolites *A* and *B* are the ‘input’ metabolites and *G* is the ‘output’ metabolite. In such a case, we can see that the network consists of three double lethal pairs: {(*R*_1_, *R*_2_), (*R*_4_, *R*_6_) and (*R*_5_, *R*_6_)}. Taking the reaction pair (*R*_4_, *R*_6_) into consideration, we can see that when reaction *R*_4_ is active, and *R*_6_ is deleted or inactive, all the fluxes will be routed through reactions *R*_4_ and *R*_5_, as shown in Fig. 9c. Similarly, when reaction *R*_6_ is active, and *R*_4_ is deleted or inactive, all the fluxes will be routed through reaction *R*_6_, as shown in Fig. 9b. In addition to these changes, the fluxes routed through the remaining reactions could vary based on which pathway is chosen. For instance, the flux through reaction *R*_3_ could be significantly higher when the *R*_4_ pathway is used than when the *R*_6_ pathway is used. We define the ‘minRerouting set’ as the minimal reaction set comprising all the reactions that have a modified flux following the individual deletion of synthetic double lethal reactions. Hence, from Fig. 9, when reaction *R*_4_ is active, and reaction *R*_6_ is inactive or deleted, the first rerouting set becomes *R*_3_, *R*_4_, and *R*_5_. When reaction *R*_6_ is active, and reaction *R*_4_ is inactive or deleted, the second rerouting set becomes *R*_3_ and *R*_6_. The common rerouting set for the double lethal pair (*R*_4_, *R*_6_) consists of *R*_3_ and the complete ‘minRerouting set’ for the double lethal pair becomes *R*_3_, *R*_4_, *R*_5_, and *R*_6_.

In order to determine the minRerouting set, we first obtain the wild-type flux distribution, **v**_WT_, and the set of all lethal pairs in the model. Then, for each lethal pair *R*_*i*_ and *R*_*j*_, the optimal flux distributions ${{\bf{v}}}_{\Delta {R}_{i}}$ and ${{\bf{v}}}_{\Delta {R}_{j}}$, that minimise the distance between the two flux distributions is obtained, by an extension of the MOMA formulation. The generalised *p*-norm formulation for obtaining the minRerouting of a metabolic network, for a given lethal pair, is as follows:

*Step 1*: An adaptation of MOMA is performed to obtain the optimal flux distributions ${{\bf{v}}}_{\Delta {R}_{i}}$ and ${{\bf{v}}}_{\Delta {R}_{j}}$ with minimal flux distance between them:

3 $$\min \,\parallel {{\bf{v}}}_{\Delta {R}_{i}}-{{\bf{v}}}_{\Delta {R}_{j}}{\parallel }_{p}$$

4 $${\rm{s.t.}}\,\,{\bf{S}}{{\bf{v}}}_{\Delta {R}_{i}}={\bf{0}};\,\,{\bf{S}}{{\bf{v}}}_{\Delta {R}_{j}}={\bf{0}}$$

5 $${{\bf{v}}}_{{{LB}}}\preccurlyeq {{\bf{v}}}_{\Delta {R}_{i}}\preccurlyeq {{\bf{v}}}_{{{UB}}};\,\,{{\bf{v}}}_{{{LB}}}\preccurlyeq {{\bf{v}}}_{\Delta {R}_{j}}\preccurlyeq {{\bf{v}}}_{{{UB}}}$$

6 $${v}_{\Delta {R}_{i},{R}_{i}}=0;\,\,{v}_{\Delta {R}_{j},{R}_{j}}=0$$

7 $${v}_{\Delta {R}_{i},{\rm{bio}}}\ge (1-\gamma ){v}_{\Delta {R}_{i},{\rm{bio}}}^{* }$$

8 $${v}_{\Delta {R}_{j},{\rm{bio}}}\ge (1-\gamma ){v}_{\Delta {R}_{j},{\rm{bio}}}^{* }$$

where ${{\bf{v}}}_{\Delta {R}_{i}}$ and ${{\bf{v}}}_{\Delta {R}_{j}}$ represent the flux distribution when reactions *R*_*i*_ and *R*_*j*_ are deleted, respectively. ${v}_{\Delta {R}_{i},{\rm {bio}}}^{* }$ and ${v}_{\Delta {R}_{j},{\rm {bio}}}^{* }$ represent the optimal biomass flux in the models when reaction *R*_*i*_ and *R*_*j*_ are deleted, respectively. ${v}_{\Delta {R}_{i},{R}_{i}}$ and ${v}_{\Delta {R}_{j},{R}_{j}}$ represent the flux through reactions *R*_*i*_ and *R*_*j*_ in the models where *R*_*i*_ and *R*_*j*_ are deleted, respectively. *γ* is the growth rate slack provided for the new flux distributions ${{\bf{v}}}_{\Delta {R}_{i}}$ and ${{\bf{v}}}_{\Delta {R}_{j}}$ from the optimal biomass ${v}_{\Delta {R}_{i},{\rm {bio}}}^{* }$ and ${v}_{\Delta {R}_{j},{\rm {bio}}}^{* }$.

*Step 2*: The flux distributions ${{\bf{v}}}_{\Delta {R}_{i}}$ and ${{\bf{v}}}_{\Delta {R}_{j}}$, obtained from Eqs. (3)–(8) are analysed. The reactions that have different flux values in ${{\bf{v}}}_{\Delta {R}_{i}}$ and ${{\bf{v}}}_{\Delta {R}_{j}}$ are identified as the *rerouting set*. The size of the rerouting set, the individual reaction flux difference and the total flux difference are subsequently analysed.

For obtaining the *L*_0_-norm solution, we used the LP formulation and IBM ILOG CPLEX v12.8 solver as it was one of the few solvers which supported *L*_0_-norm optimisation. We used the Gurobi solver for the *L*_1_-norm and *L*_2_-norm optimisations.

### Plasticity and redundancy in synthetic lethals

Previously^18^, it has been suggested that synthetic lethal reaction pairs can be classified into two categories: plastic synthetic lethal (PSL) and redundant synthetic lethal (RSL). PSL comprises of reaction pairs where one reaction acts as a backup for the other, i.e., the second reaction becomes active when the first reaction is deleted. RSL comprises reaction pairs where both reactions are active simultaneously.

The classification approach proposed by a previous study^18^ is based on flux vectors which are predicted using FBA. While an FBA solution satisfies all the flux and stoichiometric constraints for a given model, it only represents one possible flux instance from the permissible flux space. Here, we propose a classification approach that is more systematic and thorough, taking into consideration the allowable flux space for each reaction that is part of a double lethal pair. Thus, if solutions where both reactions are not active at the same time can be found, the pair is classified as a PSL.

For instance, using the above approach, if the absolute fluxes of two synthetic lethal reactions, R_1_ and R_2_, obtained from FBA, are greater than 0, then, they are classified as RSL reactions. However, there is also a chance that R_2_ can accommodate zero flux without any change in the optimal biomass flux, while R_1_ is active. In this case, the reaction pair would have to be classified as a PSL pair. As FBA only picks a single flux instance from the permissible space, we would not be able to correctly classify these reaction pairs. This necessitates a more thorough and systematic manner of classifying the reaction classes.

In order to classify the lethal pairs as PSL or RSL, we performed a flux variability analysis (FVA)^65^ on the model. FVA is used to obtain the maximum and minimum flux values that a reaction can carry in a model. It solves two LP problems (maximisation and minimisation) for each reaction in the model while constraining the objective function (or) biomass growth rate value. FVA is formulated as follows:

9 $$\min /\max \,{v}_{j}$$

10 $${{s.t.}}\,\,{\bf{S}}{\bf{v}}={\bf{0}};\,\,{{\bf{v}}}_{{{LB}}}\preccurlyeq {\bf{v}}\preccurlyeq {{\bf{v}}}_{{{UB}}};\,\,{{\bf{v}}}_{{{bio}}}={{\bf{v}}}_{{{WT,bio}}};$$

The product of the minimum and maximum flux ranges is used to determine the category of the reaction pair. In case a reaction *R*_*i*_ is always active, the product of its minimum and maximum fluxes will always be positive as 0 is not in the range of permissible flux values. When both the reactions are simultaneously active (with a positive or negative flux), while satisfying the biomass constraint, it is considered an RSL pair. The product of the sign of the minimum and maximum fluxes for RSL pairs given as [*R*_*i*_, *R*_*j*_] includes the combinations [(<0, <0), (<0, <0)], [(>0, >0), (>0, >0)], [(<0, <0), (>0, >0)], and [(>0, >0), (<0, <0)]. In cases where this is not satisfied, a conditional FVA is performed before the double lethal pair is classified as PSL or RSL.

**Fig. 10:**
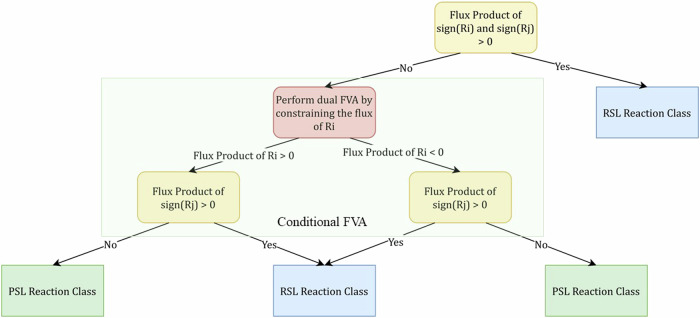
Flowchart depicting the classification of reaction pairs into PSL or RSL pairs. After the initial FVA, conditional FVAs are performed to classify the ambiguous reaction pairs. The reaction pairs are classified as RSL only when both reactions are simultaneously active.

For each of the ambiguous conditions, two conditional FVAs are performed. In the first FVA, the flux of *R*_*i*_ is constrained to be >0 and in the second, the flux of *R*_*i*_ is constrained to be <0. In this manner, the maximum and minimum fluxes of reaction *R*_*j*_ are obtained when reaction *R*_*i*_ is active. If the reaction *R*_*j*_ can carry a flux value of zero, in either of the two constraint conditions, the reaction pair is considered to be a PSL reaction pair, as *R*_*j*_ can be inactive when *R*_*i*_ is active. However, if *R*_*j*_ is always active under both constraint conditions, the reaction pair is considered to be an RSL. We used this process to determine the classification of PSL and RSL classes instead of relying on a simple FVA because, in FVA, we obtain the maximum and minimum flux values of one reaction, independent of the activity of the other. The whole process is explained pictorially in Fig. 10.

### Parsimonious FBA (pFBA)

pFBA^40^ employs a bi-level optimisation problem, where first, an optimal flux distribution that maximises the biomass production is identified, followed by the minimisation of total flux through all reactions. In addition to obtaining this flux distribution, pFBA also classifies all the reactions based on their enzymatic/metabolic efficiency. pFBA classifies reactions into a total of six classes: (i) Essential, (ii) enzymatically less efficient (ELE), (iii) metabolically less efficient (MLE), (iv) pFBA optimal, (v) blocked, and (vi) zero flux reactions. We used these reaction classes further, to classify the reactions in the lethal pairs and derive insights into the categorical distribution of these classes across lethal pairs.

### Flux redistribution by synthetic lethals

For each synthetic lethal pair, the rerouting set would comprise two subsets—one for each of the double lethal reactions, seen in Supplementary Fig. 1. The reaction sets were analysed, and the properties studied, along with their definitions, are provided below:

- Size of the synthetic lethal (SL) cluster, number of reactions with a modified flux given by the union of minRerouting subsets 1 and 2 or the complete minRerouting set
- Size of the common synthetic lethal cluster, the intersection of the two minRerouting subsets representing the reactions that remain active with a modified flux
- Net flux difference, the absolute sum of the net difference between the final flux vectors obtained using *minRerouting*
- Synthetic Accessibility (SA), the fraction of distinct reactions flux is rerouted through for a given SL pair:
  $${\rm{SA}}=\frac{{\rm{Symmetric}}\,{\rm{difference}}\,{\rm{of}}\,{\rm{the}}\,{\rm{subsets}}}{{\rm{Complete}}\,{\rm{minRerouting}}\,{\rm{set}}}$$
- Redundancy index (RI), the fraction of synthetic lethal pairs in which a reaction (*R*_*i*_) occurs within a species:
  $${\rm{RI}}=\frac{{\rm{Number}}\,{\rm{of}}\,{\rm{synthetic}}\,{\rm{lethal}}\,{\rm{pairs}}\,{R}_{i}\,{\rm{is}}\,{\rm{part}}\,{\rm{of}}}{{\rm{Total}}\,{\rm{number}}\,{\rm{of}}\,{\rm{synthetic}}\,{\rm{lethal}}\,{\rm{pairs}}}$$
- Reaction compensation index (RCI), the fraction of synthetic clusters in which a reaction (*R*_*i*_) occurs within a species:
  $${\rm{RCI}}=\frac{{\rm{Number}}\,{\rm{of}}\,{\rm{synthetic}}\,{\rm{lethal}}\,{\rm{clusters}}\,{R}_{i}\,{\rm{is}}\,{\rm{part}}\,{\rm{of}}}{{\rm{Total}}\,{\rm{number}}\,{\rm{of}}\,{\rm{synthetic}}\,{\rm{lethal}}\,{\rm{clusters}}}$$

### Changing the environment of organisms

The media was changed to depict the change in the environment faced by an organism by fixing the lower bound concentrations of the exchange reactions. First, we changed the concentration of 10 carbon source exchange reactions in each model to—1000 mmol g DW^−1^ h^−1^. The carbon sources are sucrose, glucose, glycerol, melibiose, cellobiose, alpha-galactose, beta-galactose, arabinose, trehalose, and fructose. These models were hence known as Model_carbon for each species. Second, we looked at the effect of unblocking all exchange reactions in the model to varying degrees, i.e., 100 mmol g DW−1 h−1, 500 mmol g DW−1 h−1, and 1000 mmol g DW^−1^ h^−1^. These models were named Model_exchange_1, Model_exchange_2, and Model_exchange_3. In total, four new models were generated for each species with different environments, and the *minRerouting* analysis was repeated for them.

### Metabolic subsystem analysis

A set of reactions that share a similar metabolic function is referred to as a metabolic subsystem^66^. All the reactions in a genome-scale metabolic model (GSMM) are categorised under different metabolic subsystems. To identify the metabolic subsystem of the reactions that comprise a double lethal pair, the set of all distinct reactions to be analysed is obtained. Then, the subsystem of each of these reactions is obtained by systematically querying the BiGG database^25^.

### Implementation

The implementation of *minRerouting* and initial analysis were done using MATLAB. The metabolic cost analysis and flux rerouting analysis were done using Python and R. The figures were generated using R. The COBRA Toolbox^67^ for MATLAB was used for all metabolic network analysis. All code written as a part of this project is open-sourced and can be accessed at https://github.com/RamanLab/minRerouting/.

## Supplementary information

Supplementary Information

## Data Availability

All models analysed during the study are taken from the BiGG database^25^.

## Code availability

The ‘minRerouting’ algorithm is available from https://github.com/RamanLab/minRerouting.
