# Supplementary material for: Understanding flux switching in metabolic networks through an analysis of synthetic lethals
Source: NPJ Syst Biol Appl. 2024 Sep 17;10:104. doi: 10.1038/s41540-024-00426-5 (PMC11408705; doi:10.1038/s41540-024-00426-5)
Supplement: Supplementary file 1 — Supplementary Information [file 41540_2024_426_MOESM1_ESM.pdf]

Understanding Flux Switching in Metabolic Networks Through an Analysis of Synthetic Lethals

Sowmya Manojna Narasimha[1,2,3,5†], Tanisha Malpani[1,3†],
Omkar S. Mohite[1,3,6], Saketha Nath[4], Karthik Raman[1,2,3*]

[1]Centre for Integrative Biology and Systems mEdicine (IBSE), Indian Institute of Technology (IIT) Madras, Chennai, 600 036, India.
[2]Department of Data Science and AI, Wadhwani School of Data Science and AI (WSAI), Indian Institute of Technology (IIT) Madras, Chennai, 600 036, India.
[3]Department of Biotechnology, Bhupat Jyoti Mehta School of Biosciences, Indian Institute of Technology (IIT) Madras, Chennai, 600 036, India.
[4]Department of Computer Science and Engineering, Indian Institute of Technology (IIT) Hyderabad, Hyderabad, 502 284, India.
[5]Present address: Neuroscience Graduate Program, University of California San Diego, San Diego, 92092, USA.
[6]Novo Nordisk Foundation Center for Biosustainability, Technical University of Denmark, 2800 Kgs. Lyngby, Denmark.

[*]Corresponding author(s). E-mail(s): kraman@iitm.ac.in;
[†]These authors contributed equally to this work.

Contents

1 Supplementary Notes 1

2 Supplementary Figures 1

3 Supplementary Tables 20

1 Supplementary Notes

The Supplementary Figures and Tables are given below. The code to generate the figures is available at `https://github.com/RamanLab/minRerouting`. Please read the main text to interpret the below analysis.

2 Supplementary Figures

The reaction distribution is explained pictorially in Supplementary Figure 1.

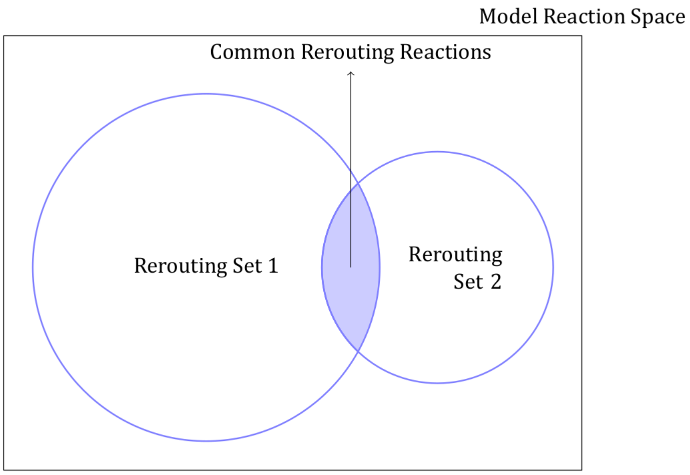

Supplementary Figure 1: **Representation of the Reaction Space and the different Rerouting Sets.** The synthetic accessibility (SA) is defined as proportion between the symmetric difference of the sets and the union of the sets.

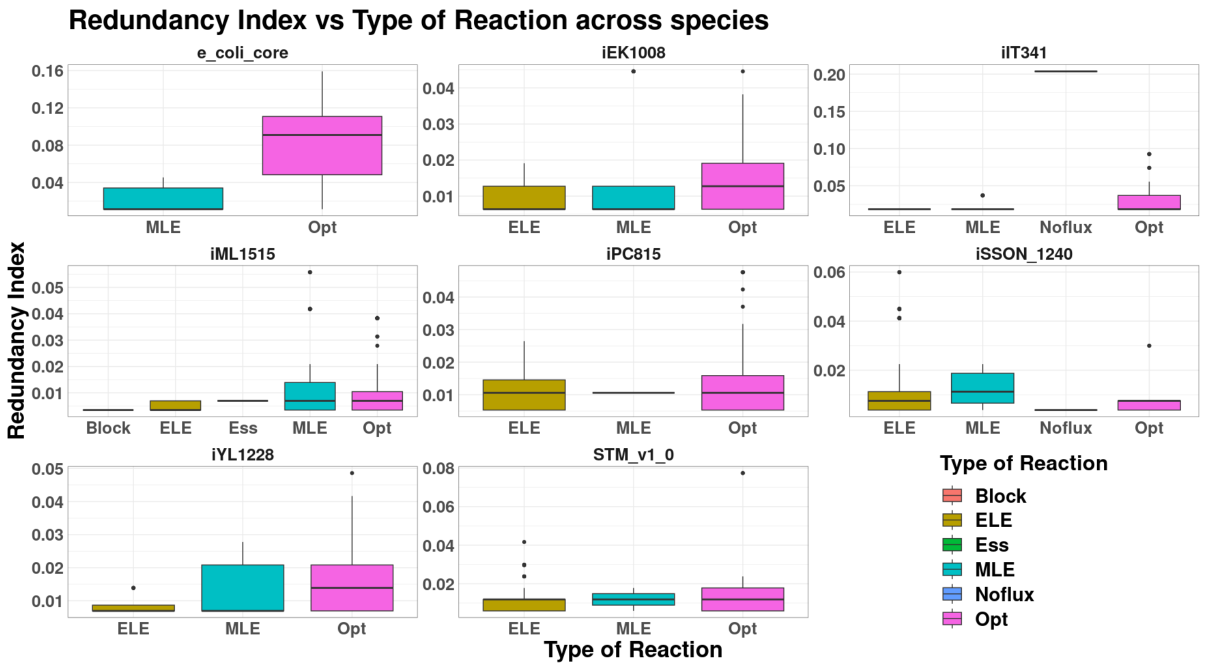

Supplementary Figure 2: **Redundancy Index of Reactions distributed over the type of reaction for each species.** There is no relation between the type of reaction and its tendency to form redundant pairs.

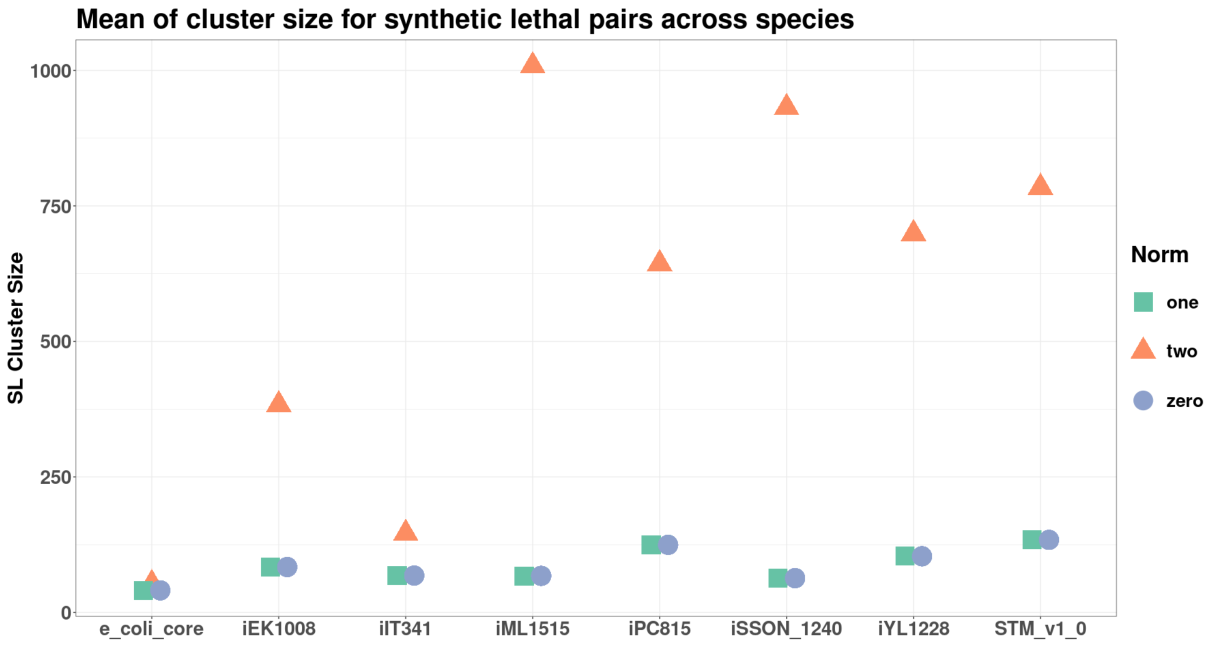

Supplementary Figure 3: **Distribution of SL Cluster Size across Organisms and Norms.** Notice that the SL Cluster Size is the smallest for the zero and one norms, while the SL Cluster Size obtained using two norm is approximately an order of magnitude higher.

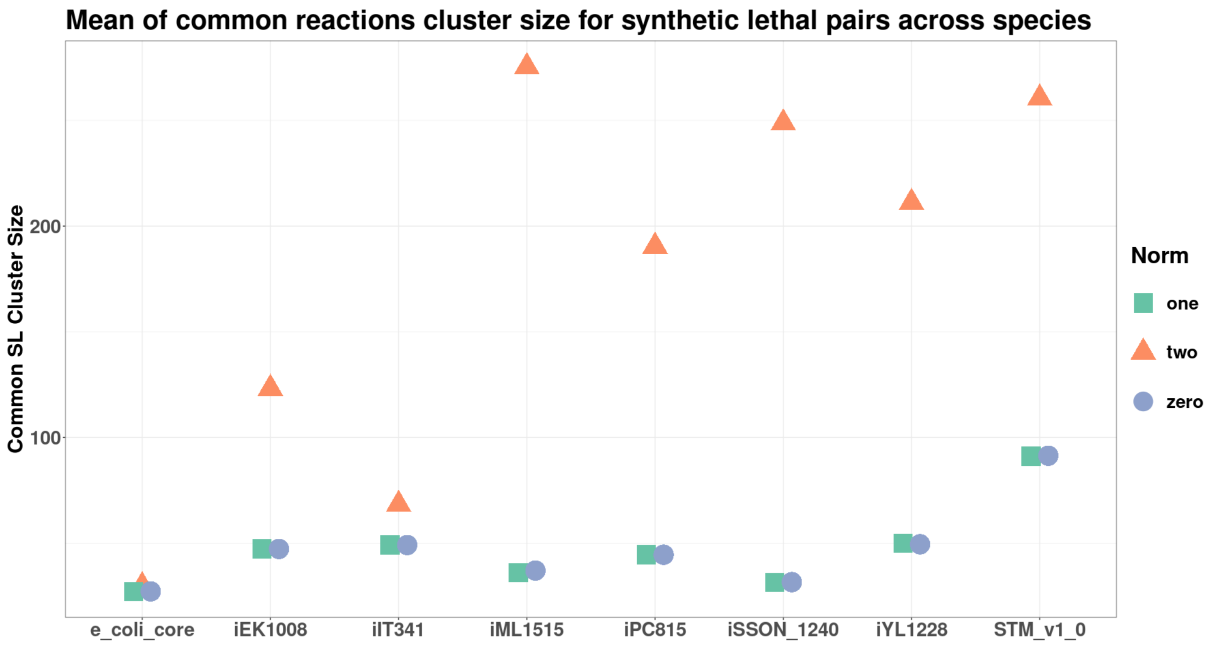

Supplementary Figure 4: **Distribution of Common SL Cluster Size across Organisms and Norms.** Notice that the Common SL Cluster Size is the smallest for the zero and one norms, while the Common SL Cluster Size obtained using two norm is approximately an order of magnitude higher.

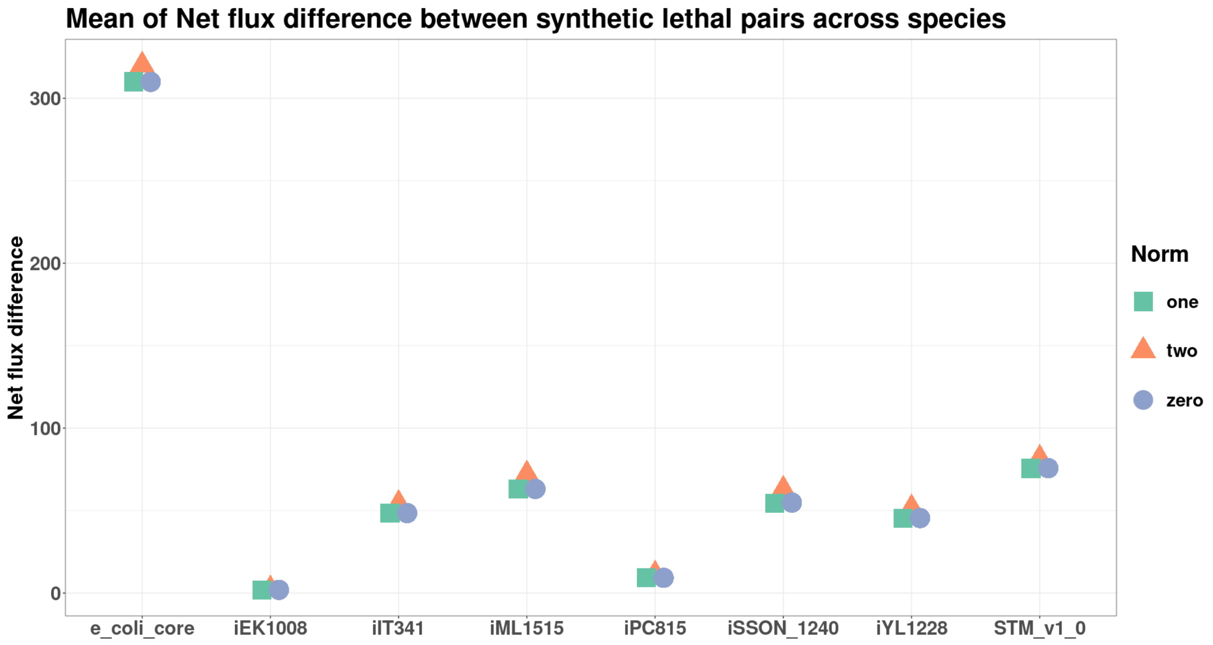

Supplementary Figure 5: **Distribution of Net flux difference between reaction pairs, across Organisms and Norms.** The net flux difference shows similar results across the norms.

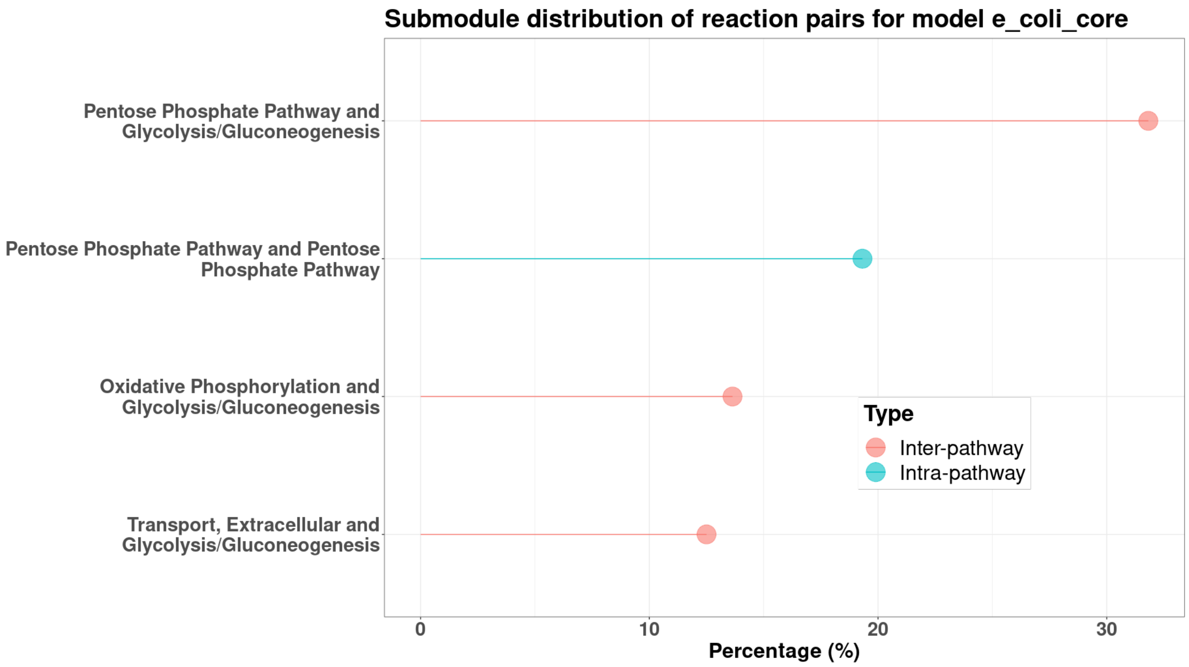

Supplementary Figure 6: **Metabolic Subsystem Analysis for the model e_coli_core.** Distribution of DLs.

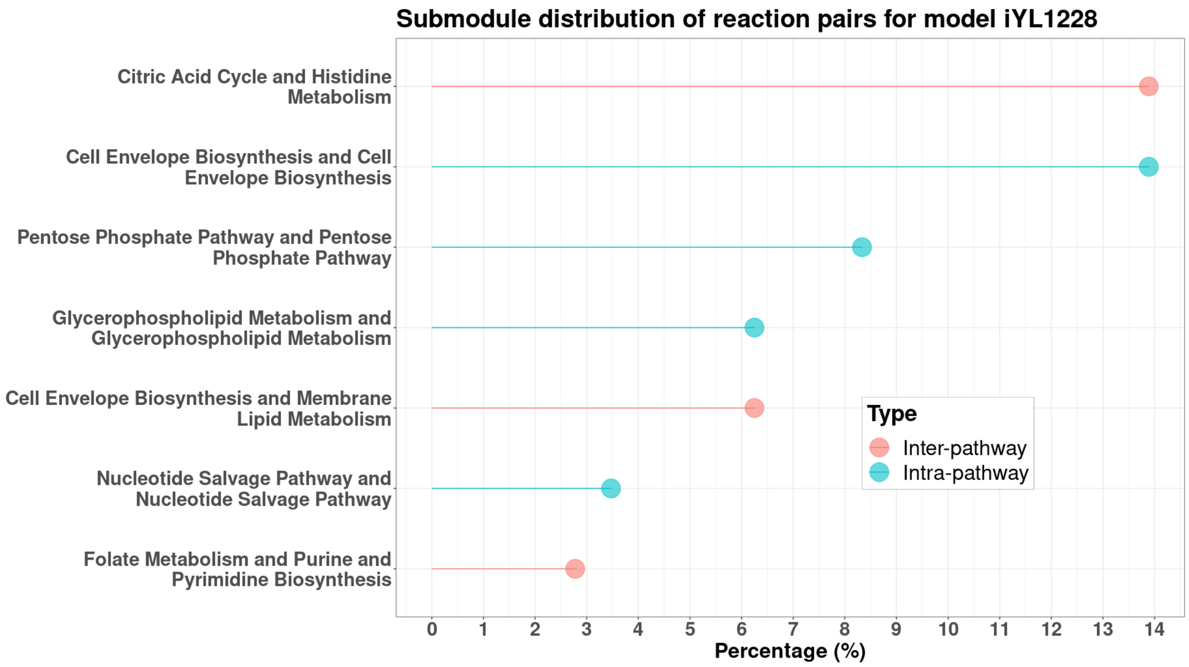

Supplementary Figure 7: **Metabolic Subsystem Analysis for the model *i*YL1228.** Distribution of DLs.

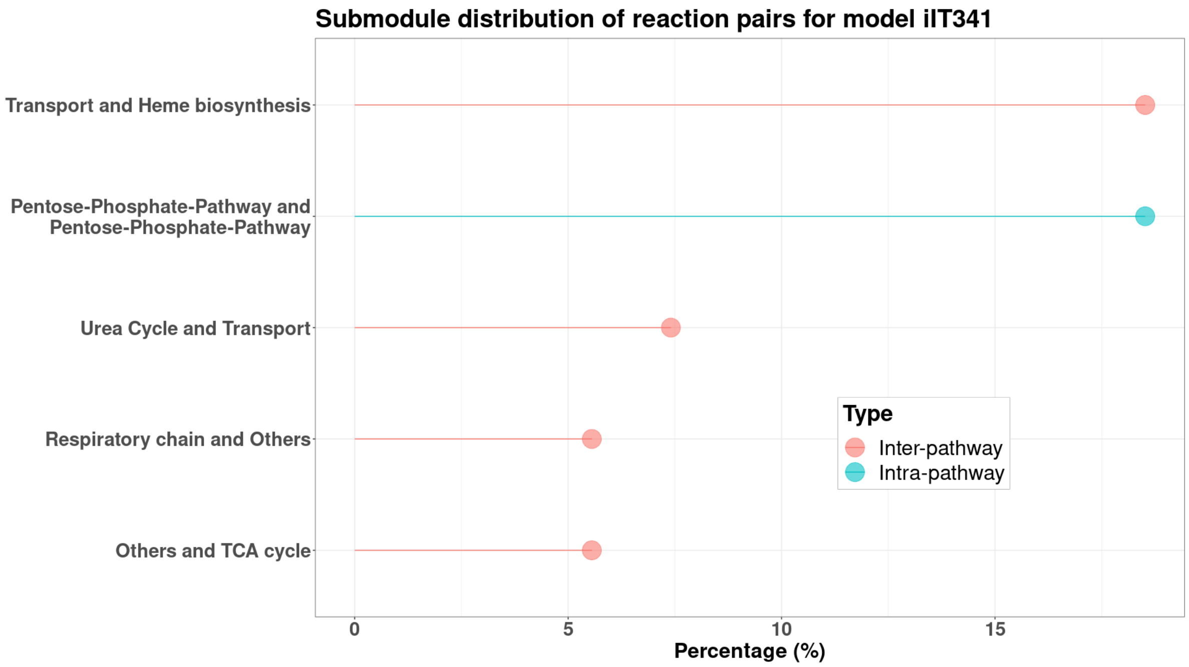

Supplementary Figure 8: **Metabolic Subsystem Analysis for the model *i*IT341.** Distribution of DLs.

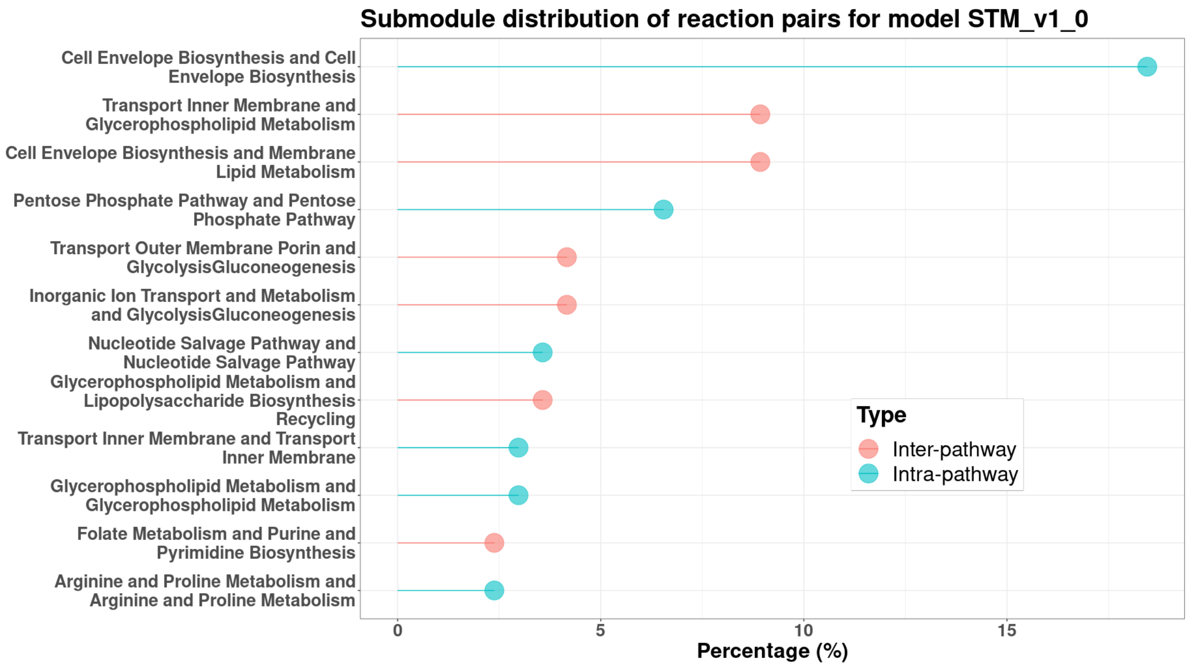

Supplementary Figure 9: **Metabolic Subsystem Analysis for the model STM_v1_0.** Distribution of DLs.

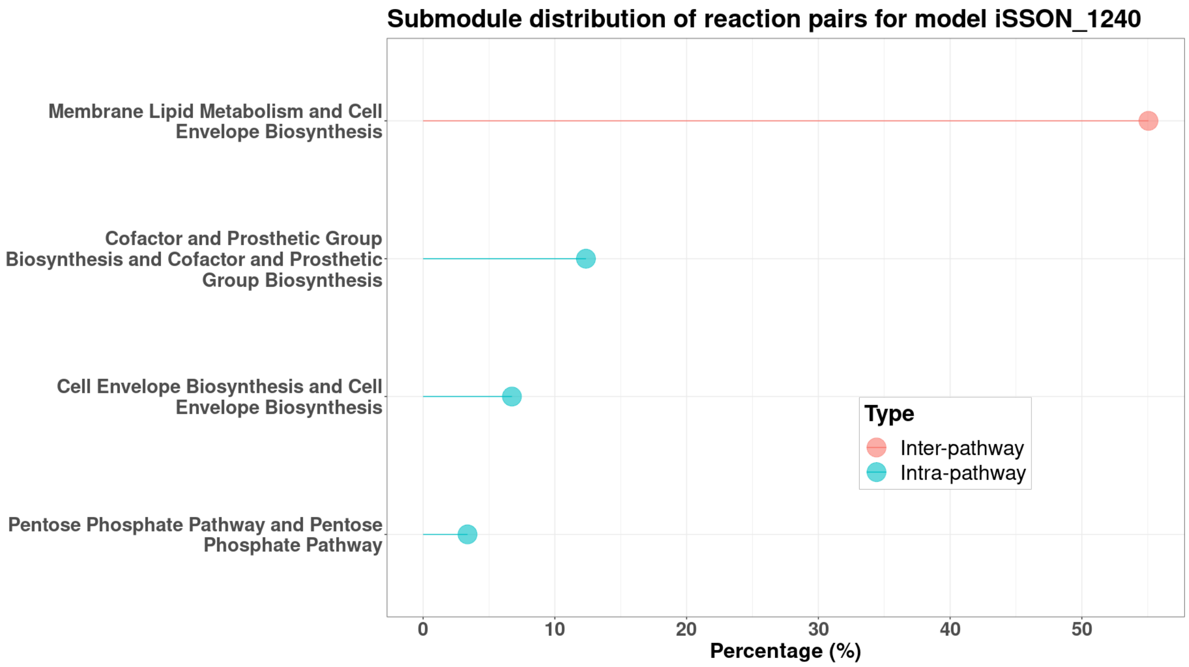

Supplementary Figure 10: **Metabolic Subsystem Analysis for the model *i*SSON_1240.** Distribution of DLs.

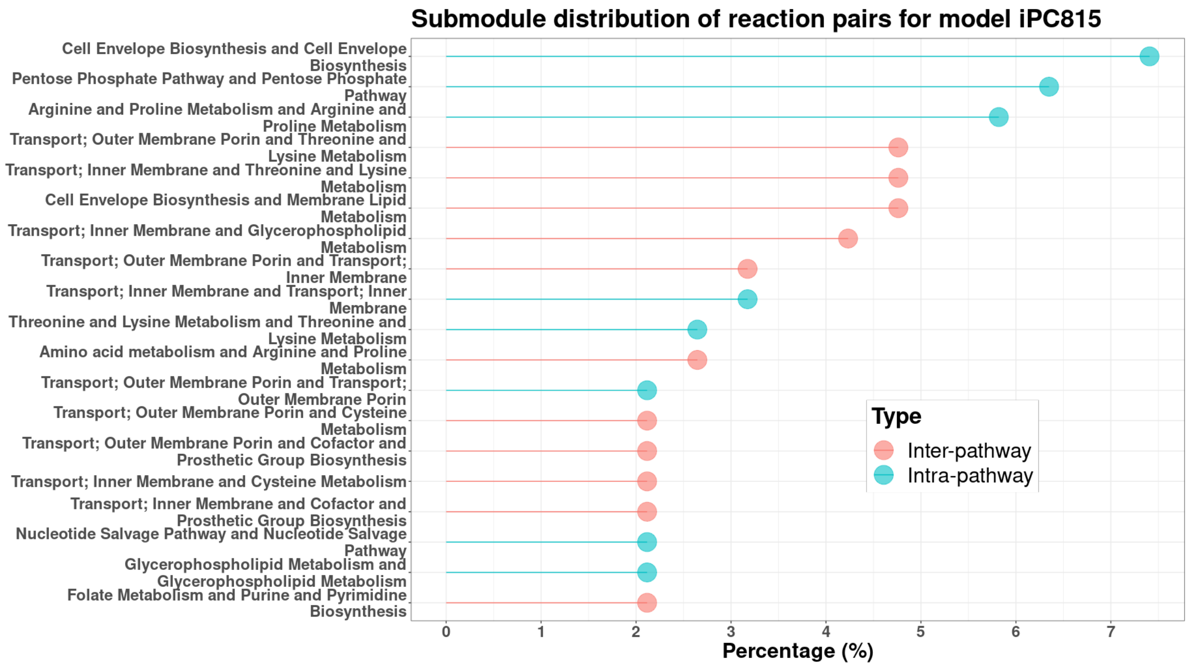

Supplementary Figure 11: **Metabolic Subsystem Analysis for the model iPC815.** Distribution of DLs.

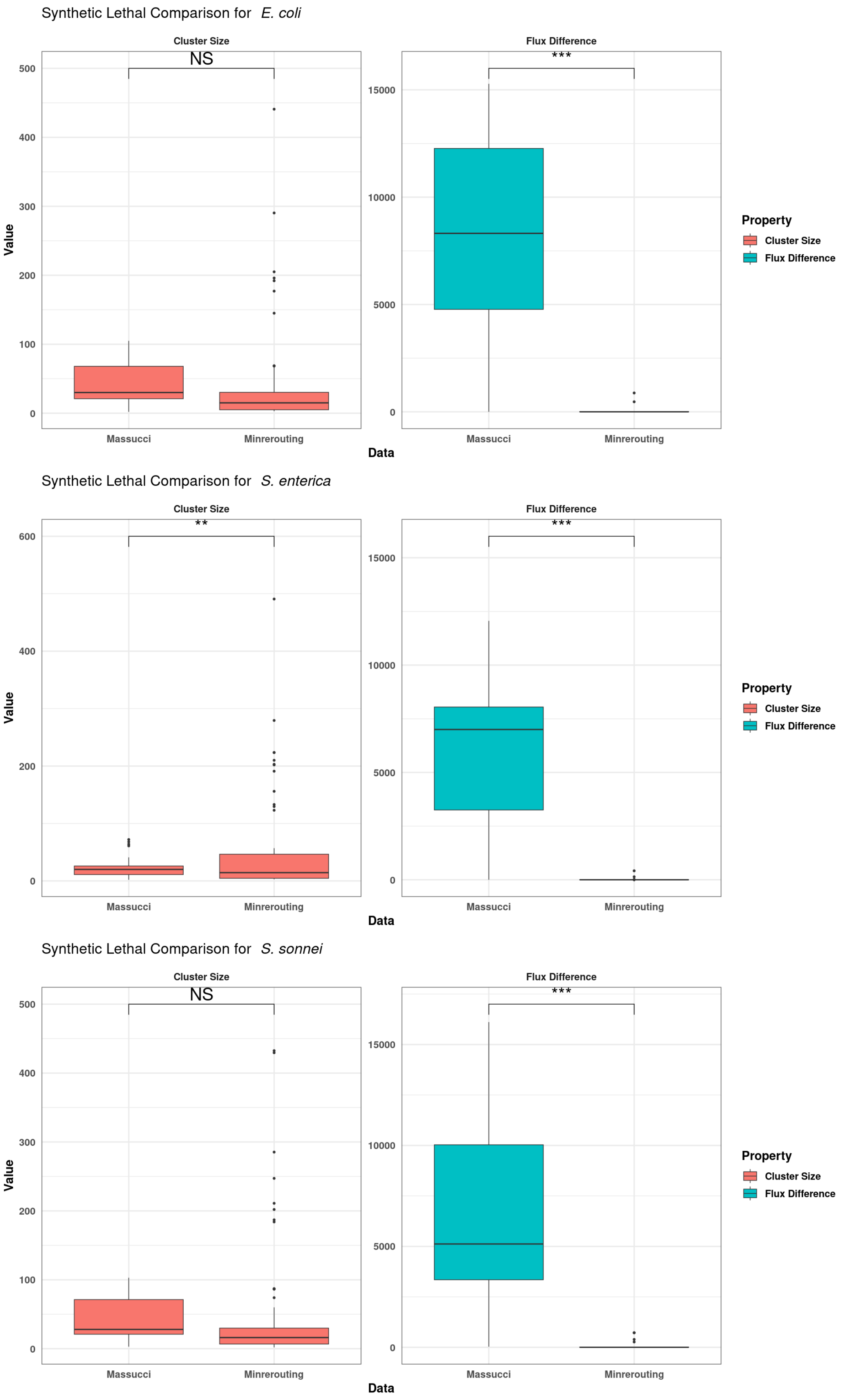

Supplementary Figure 12: **Comparison of Cluster Size and Net Flux Difference of Clusters for two different approaches to Synthetic Lethals—minRerouting and Massucci [1].** Using *minRerouting*, we calculated the flux vectors for when each reaction was active in an SL pair. Thus, we obtained our minRerouting set as all the reactions which had a change in them and then calculated flux difference. Massucci *et al.* [1], defined their cluster as the number of reactions that go from active to inactive or vice versa, and a change in flux without changing the activity of the reaction was not taken into account. Thus, net flux difference was the flux difference between the inactive and active forms of the reaction. Notice that the net flux difference is significantly lesser for minRerouting than for the observations made with a different approach. [Two-sided t-test was conducted. Significance levels: *** implies p-value < 0.001, ** implies p-value < 0.01, * implies p-value < 0.05]

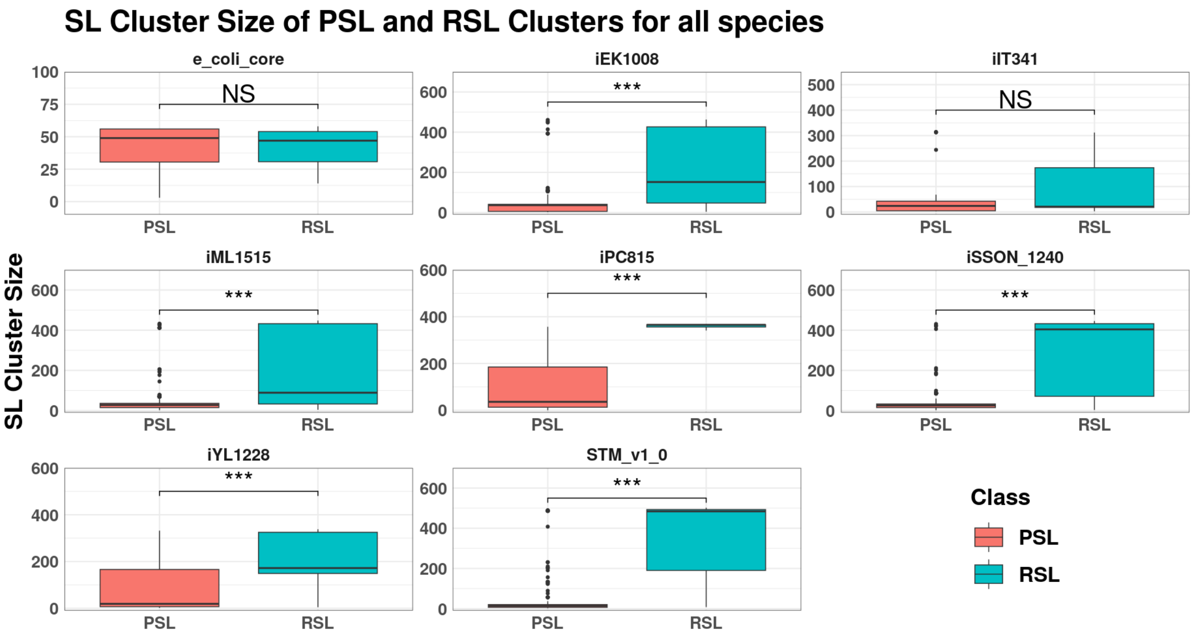

Supplementary Figure 13: **Distribution of Size of Clusters, across Organisms and Classes of the Cluster.** The size is greater for RSLs than for PSLs. [Two-sided t-test was conducted. Significance levels: *** implies p-value < 0.001, ** implies p-value < 0.01, * implies p-value < 0.05]

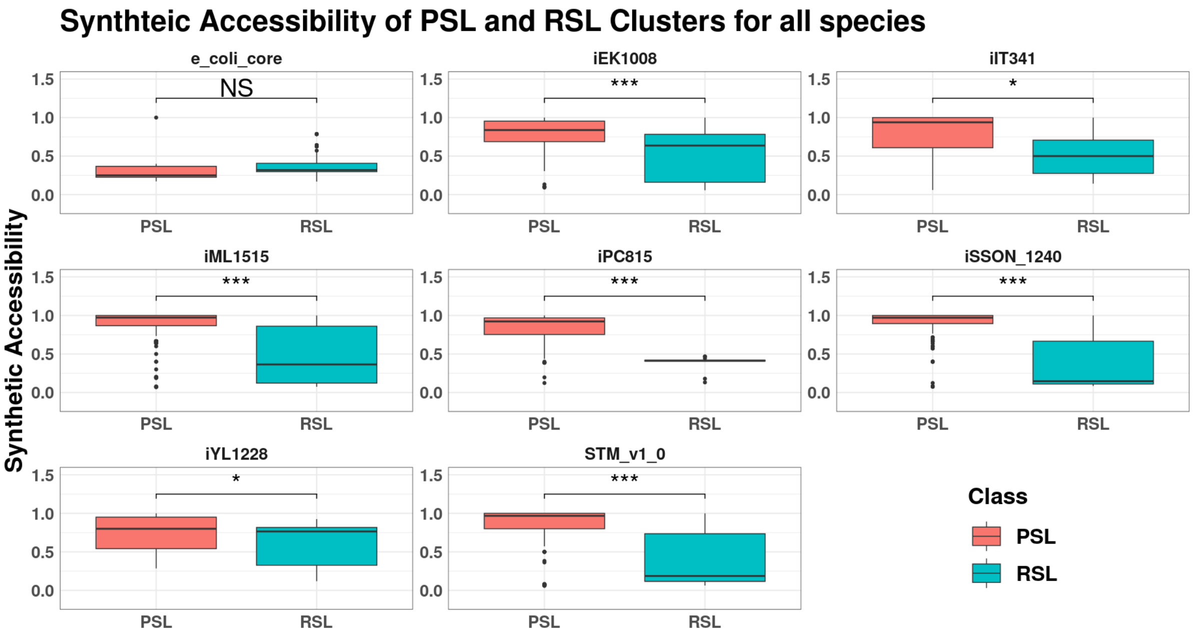

Supplementary Figure 14: **Distribution of Synthetic Accessibility of SL pairs, across Organisms and Classes of the Cluster.** The Synthetic Accessibility is smaller for RSLs than for PSLs as PSLs activate new reactions while switching fluxes. [Two-sided t-test was conducted. Significance levels: *** implies p-value < 0.001, ** implies p-value < 0.01, * implies p-value < 0.05]

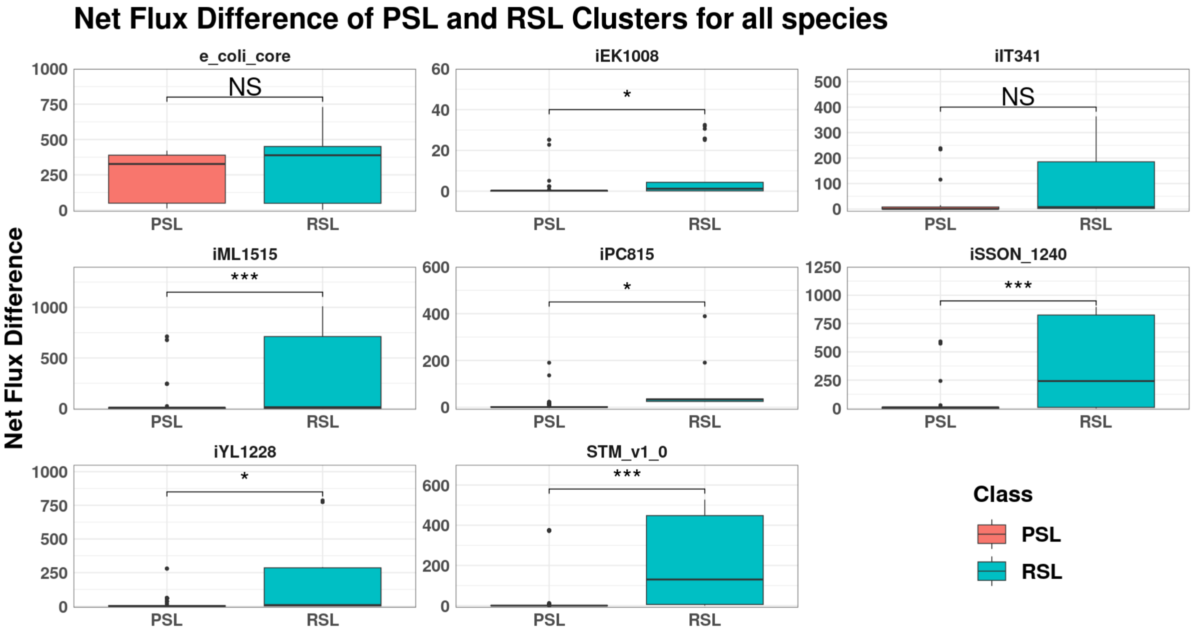

Supplementary Figure 15: **Distribution of Net Flux Difference of SL pairs, across Organisms and Classes of the Cluster.** The net flux difference is also higher for RSLs than for PSLs. [Two-sided t-test was conducted. Significance levels- *** implies p-value < 0.001, ** implies p-value < 0.01, * implies p-value < 0.05]

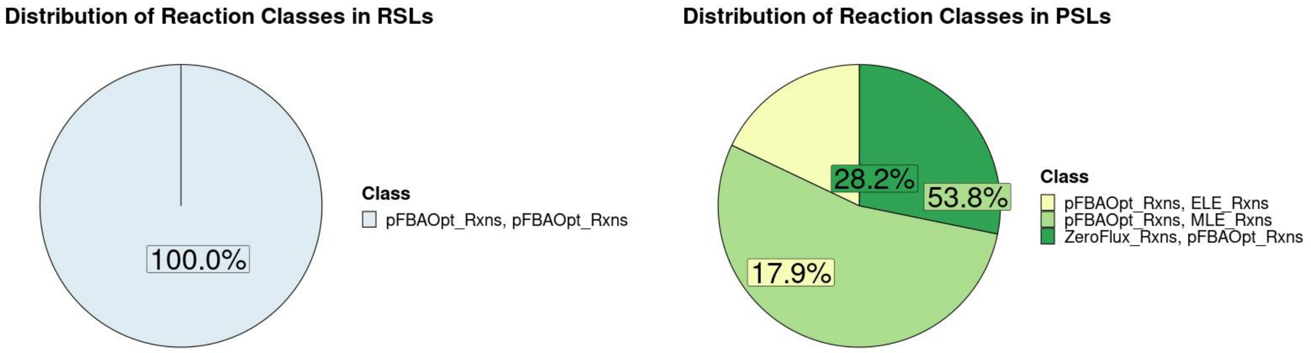

Supplementary Figure 16: **Schematic of reaction pair distribution between the RSL and PSL classes for *i*IT341.**

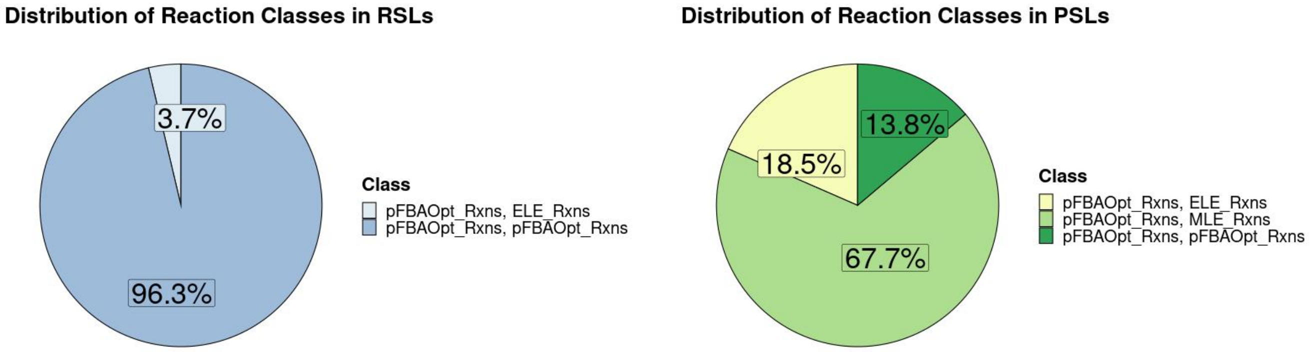

Supplementary Figure 17: **Schematic of reaction pair distribution between the RSL and PSL classes for *i*EK1008.**

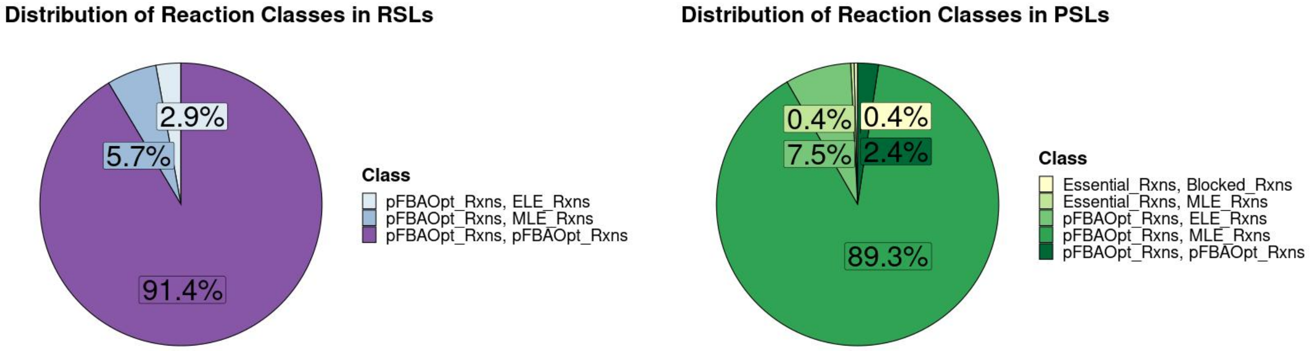

Supplementary Figure 18: **Schematic of reaction pair distribution between the RSL and PSL classes for *i*ML1515.**

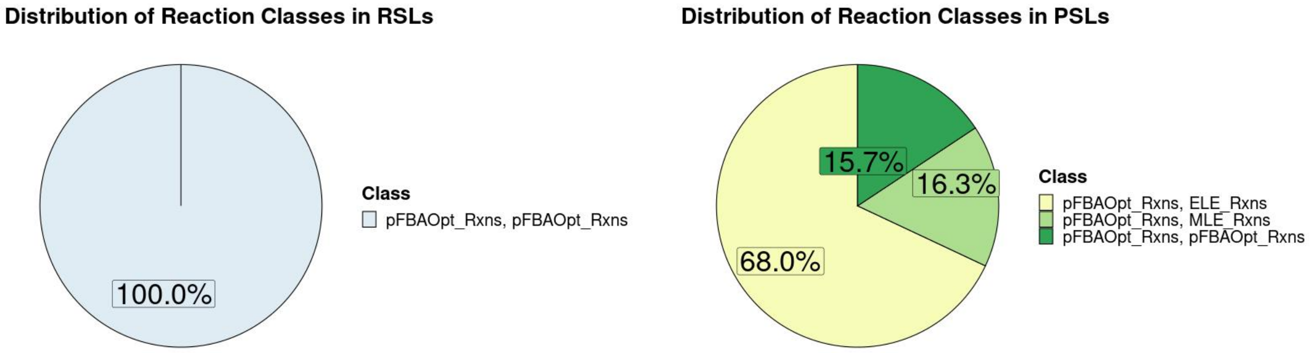

Supplementary Figure 19: **Schematic of reaction pair distribution between the RSL and PSL classes for *i*PC815.**

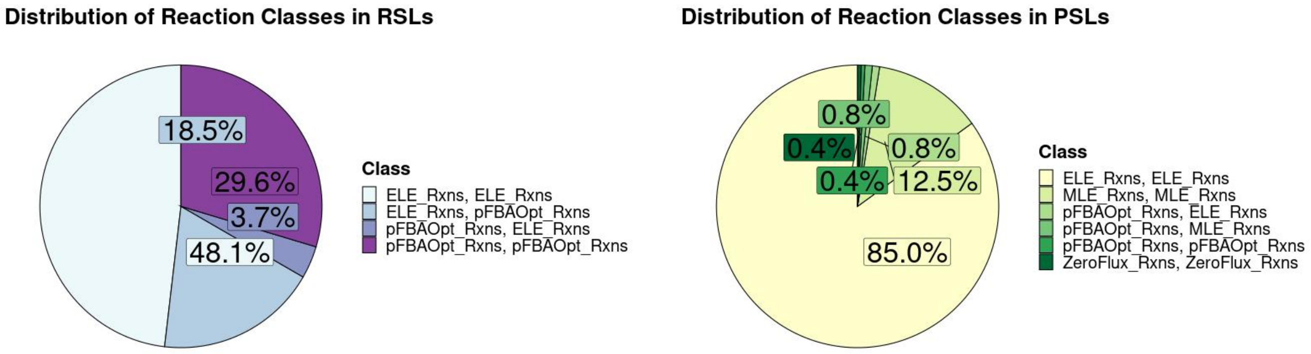

Supplementary Figure 20: **Schematic of reaction pair distribution between the RSL and PSL classes for *i*SSON_1240.**

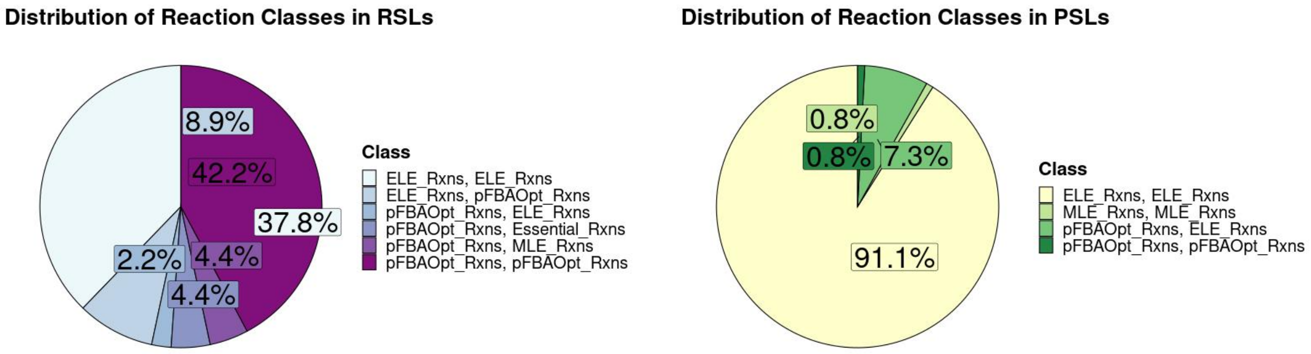

Supplementary Figure 21: **Schematic of reaction pair distribution between the RSL and PSL classes for STM_v1_0.**

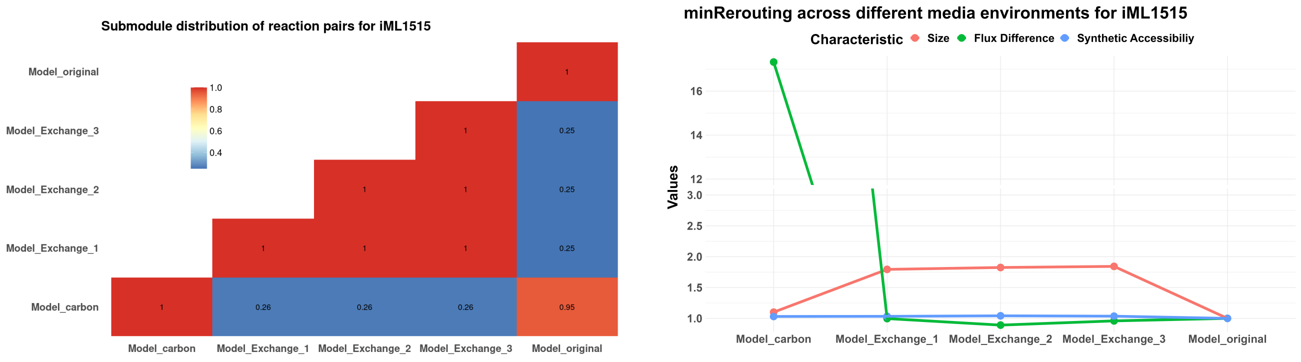

(a) (b)

Supplementary Figure 22: **Effect of media change on synthetic lethals and rerouting for the model *i*ML1515.** (a) represents the fraction overlap of double lethal reactions between models with different media conditions. (b) represents the change in flux redistribution properties as compared to original.

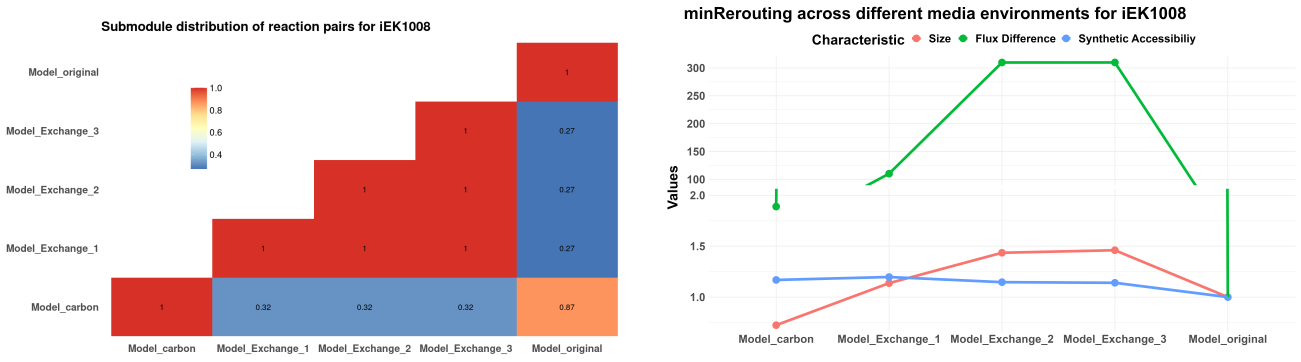

(a) (b)

Supplementary Figure 23: **Effect of media change on synthetic lethals and rerouting for the model *i*EK1008.** (a) represents the fraction overlap of double lethal reactions between models with different media conditions. (b) represents the change in flux redistribution properties as compared to original.

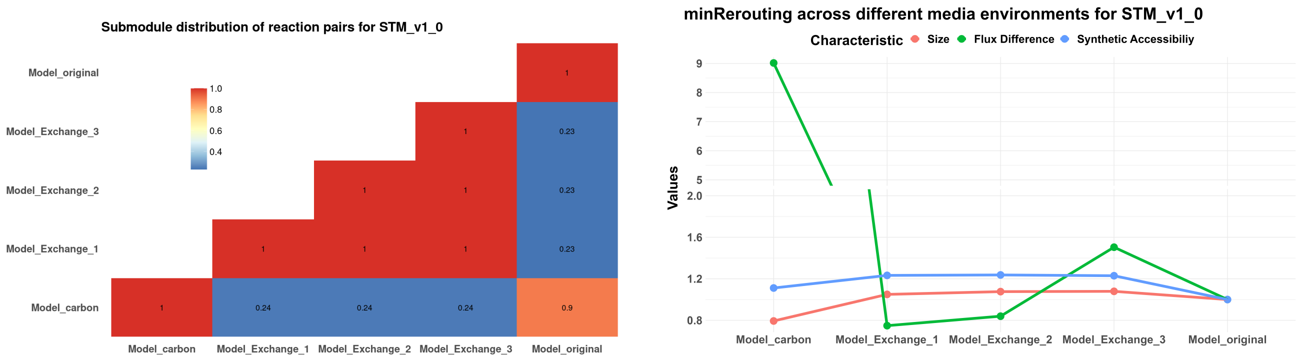

(a) (b)

Supplementary Figure 24: **Effect of media change on synthetic lethals and rerouting for the model STM_v1_0.** (a) represents the fraction overlap of double lethal reactions between models with different media conditions. (b) represents the change in flux redistribution properties as compared to original.

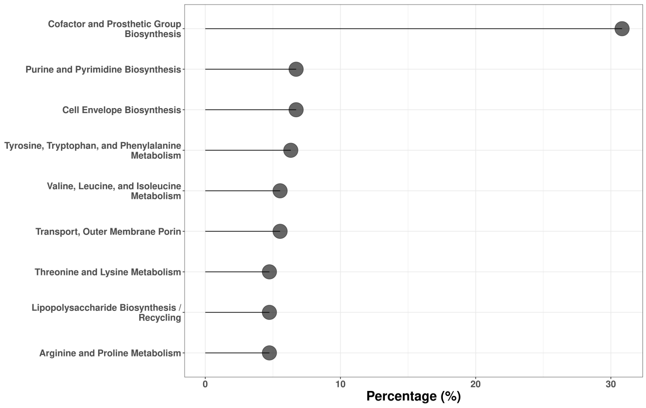

Submodule distribution of SLs for Model_carbon

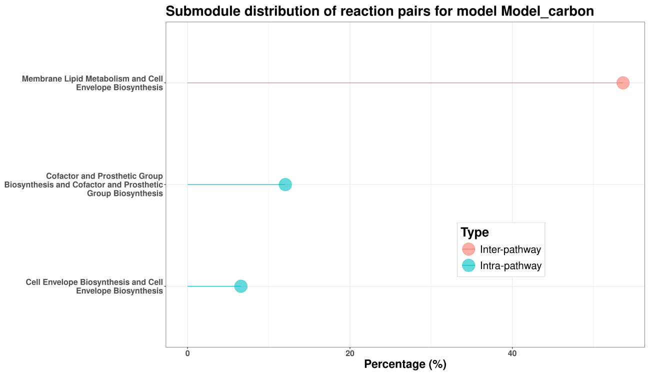

Submodule distribution of DLs for Model_carbon.

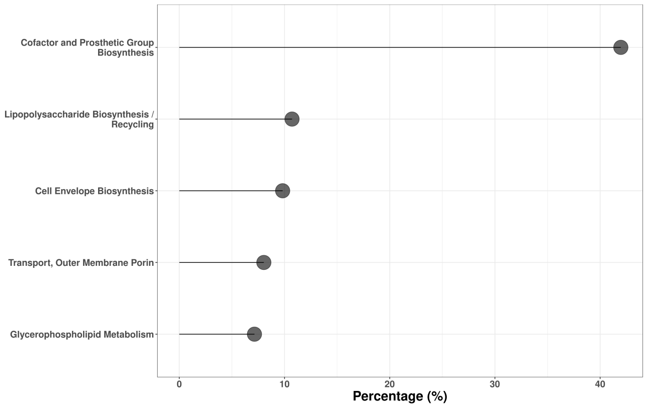

Submodule distribution of SLs for Model_exchange_1.

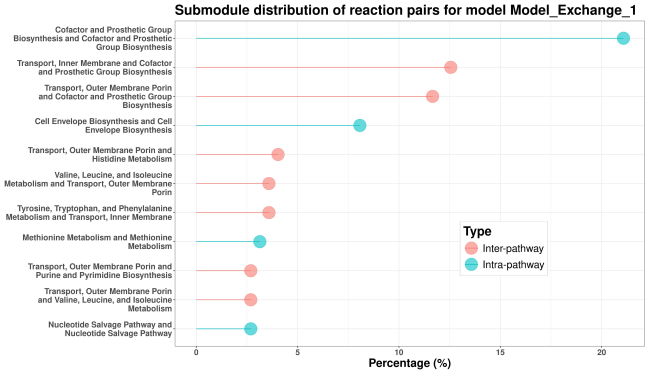

Submodule distribution of DLs for Model_exchange_1.

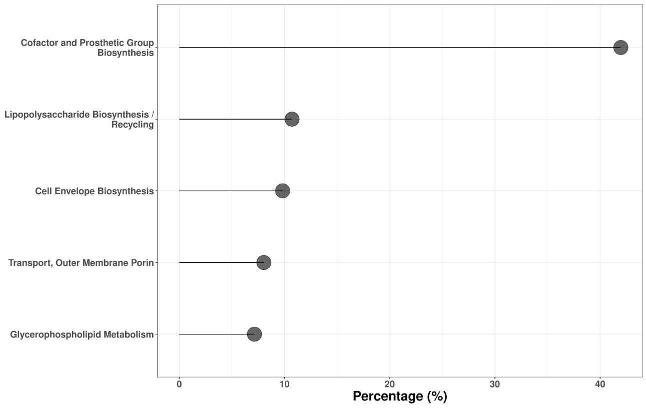

Submodule distribution of SLs for Model_exchange_3.

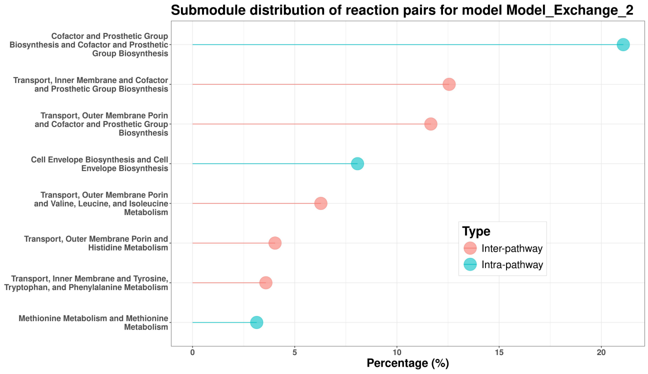

Submodule distribution of DLs for Model_exchange_3.

Supplementary Figure 25: **Effect of media change on synthetic lethals and rerouting for the model *i*ML1515.**

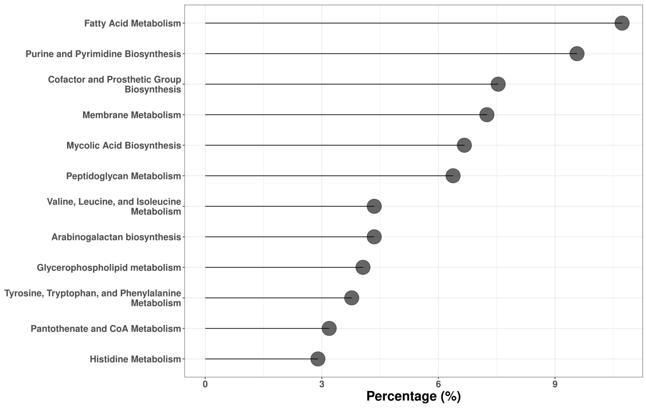

Submodule distribution of SLs for Model_carbon

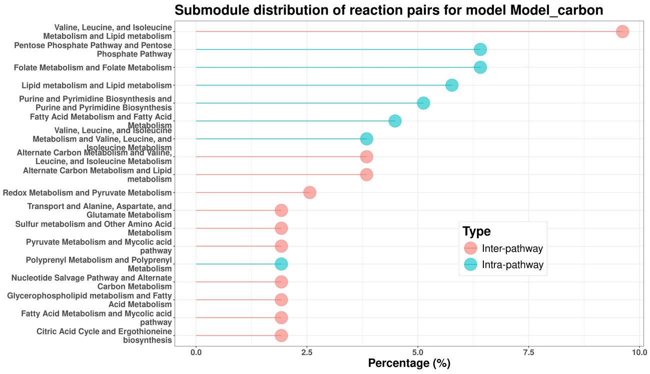

Submodule distribution of DLs for Model_carbon.

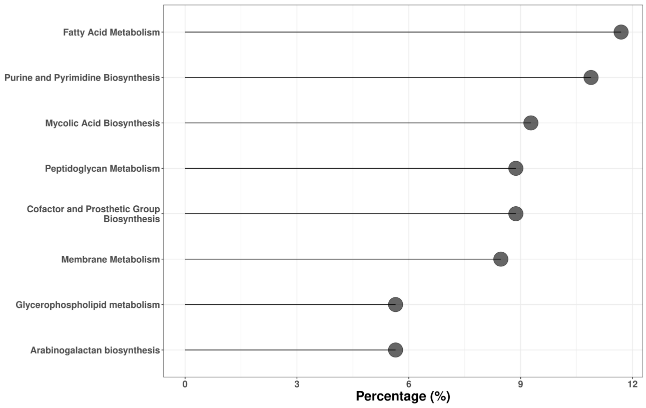

Submodule distribution of SLs for Model_exchange_1.

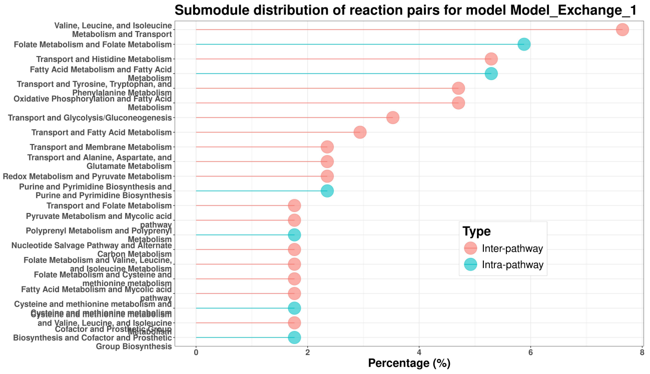

Submodule distribution of DLs for Model_exchange_1.

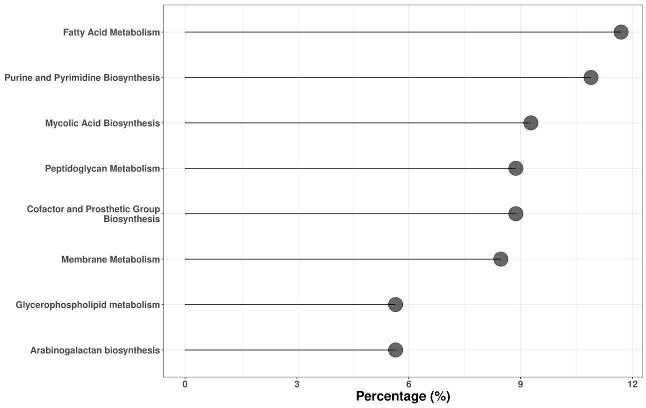

Submodule distribution of SLs for Model_exchange_3.

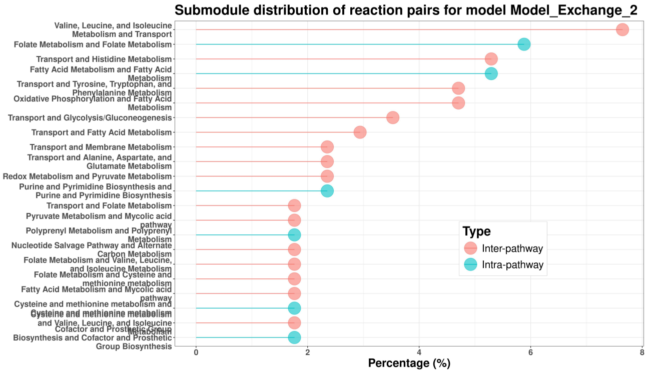

Submodule distribution of DLs for Model_exchange_3.

Supplementary Figure 26: **Effect of media change on synthetic lethals and rerouting for the model *i*EK1008.**

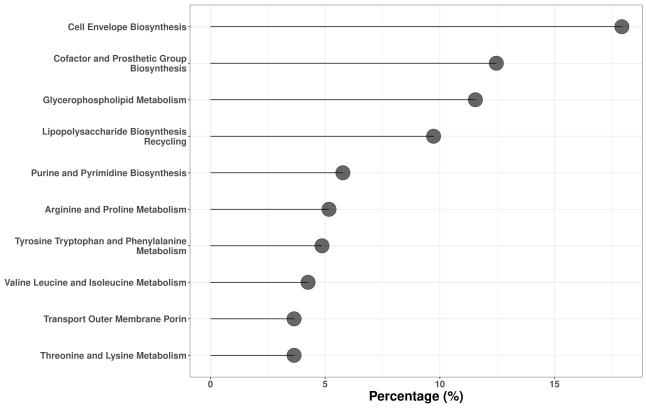

Submodule distribution of SLs for Model_carbon

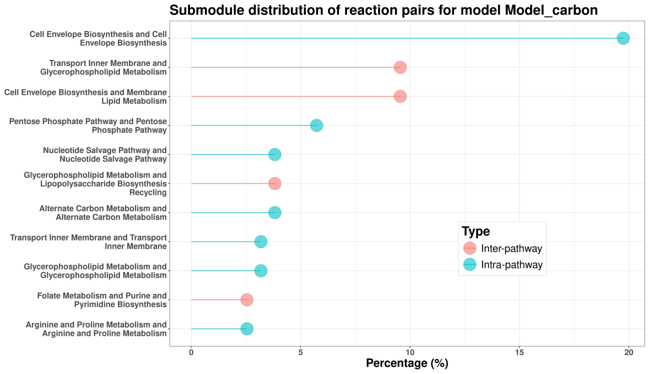

Submodule distribution of DLs for Model_carbon.

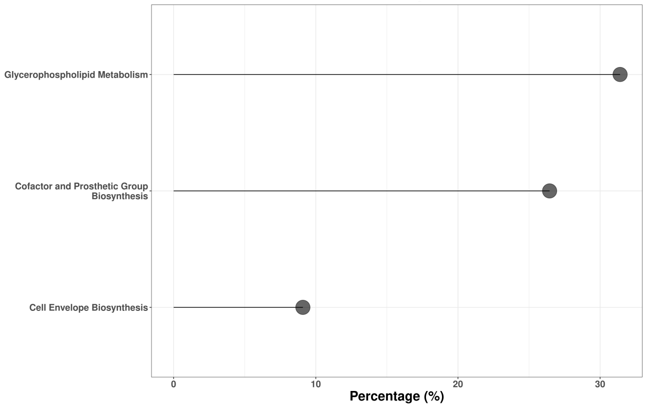

Submodule distribution of SLs for Model_exchange_1.

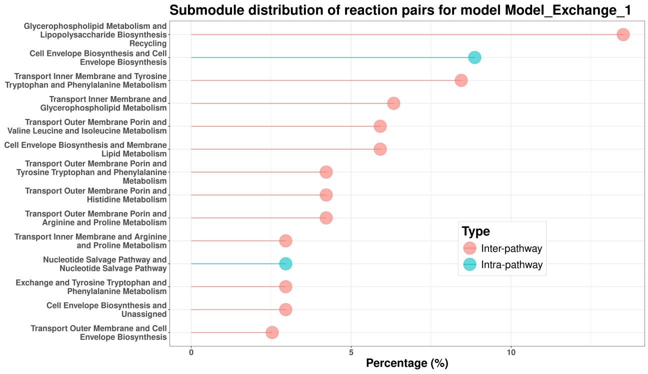

Submodule distribution of DLs for Model_exchange_1.

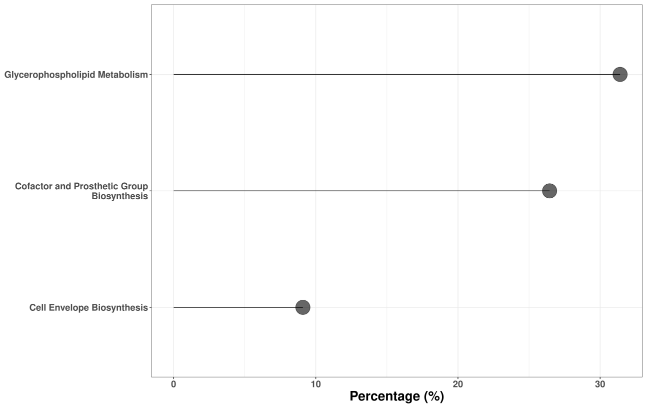

Submodule distribution of SLs for Model_exchange_3.

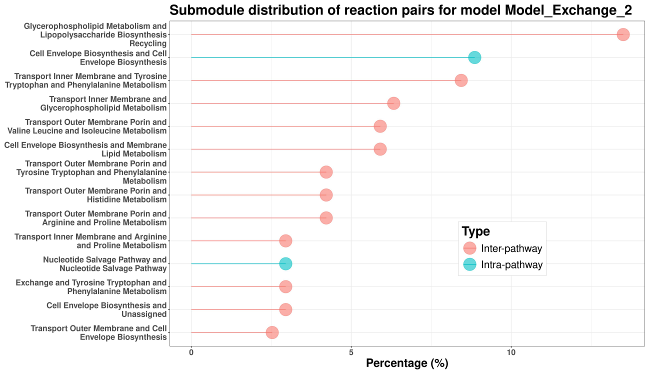

Submodule distribution of DLs for Model_exchange_3.

Supplementary Figure 27: **Effect of media change on synthetic lethals and rerouting for th emodel STM_v1_0.**

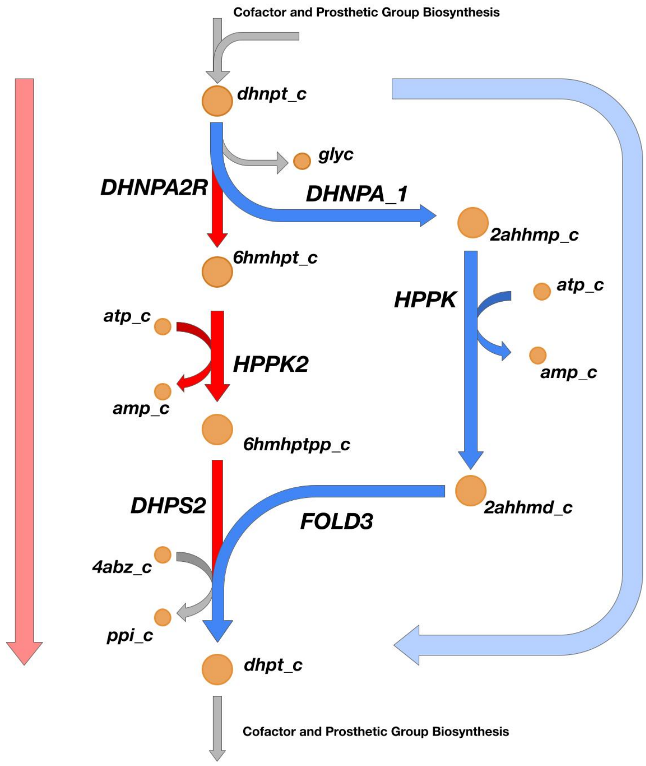

Supplementary Figure 28: **Representation of rerouting in SL pair in *i*EK1008**. The PSL pair represented is DHPS2 and FOLD3. The red and blue arrows represent the two pathways. The yellow spheres represent the metabolites and arrows represent the direction of flow of flux. Grey arrows represent common reactions.

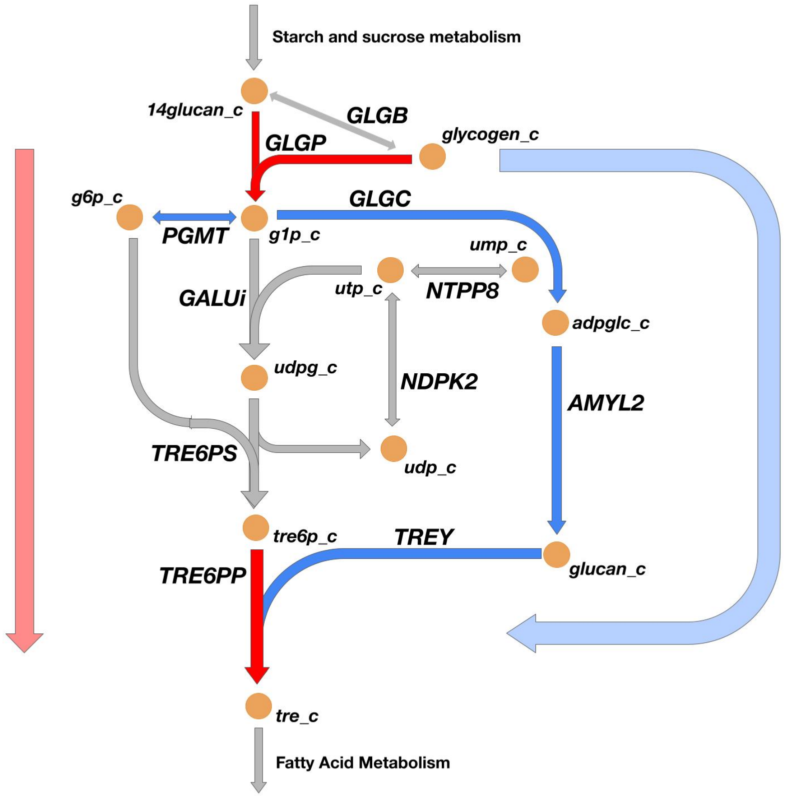

Supplementary Figure 29: **Representation of rerouting in SL pair in *i*EK1008**. The PSL pair represented is TRE6PP and TREY. The red and blue arrows represent the two pathways. The yellow spheres represent the metabolites and arrows represent the direction of flow of flux. Grey arrows represent common reactions.

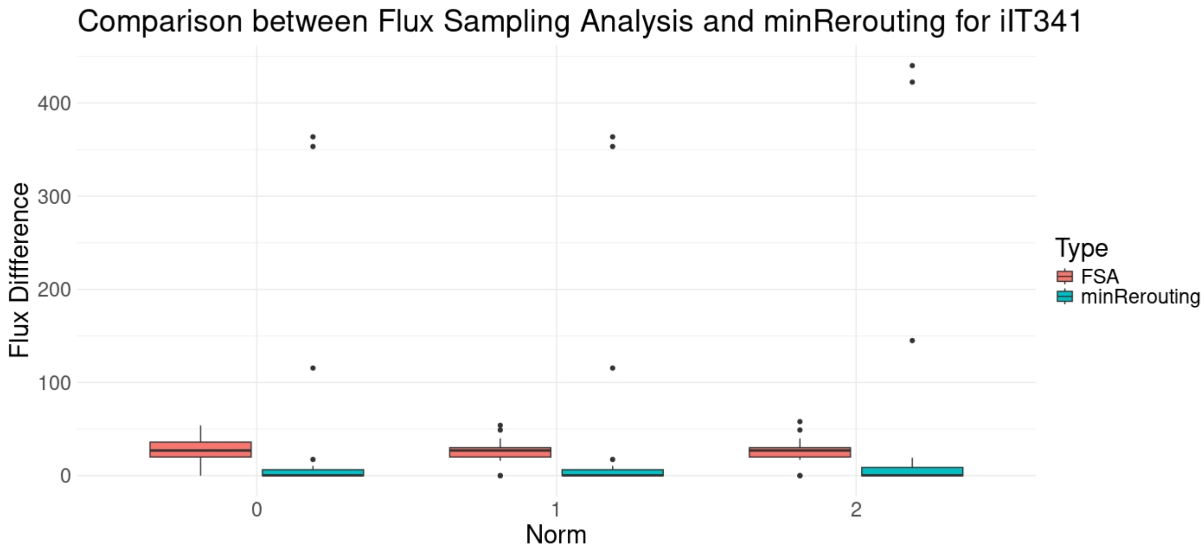

Supplementary Figure 30: **Comparison between flux sampling and minRerouting for 25 pairs of synthetic lethals for *i*IT341.**

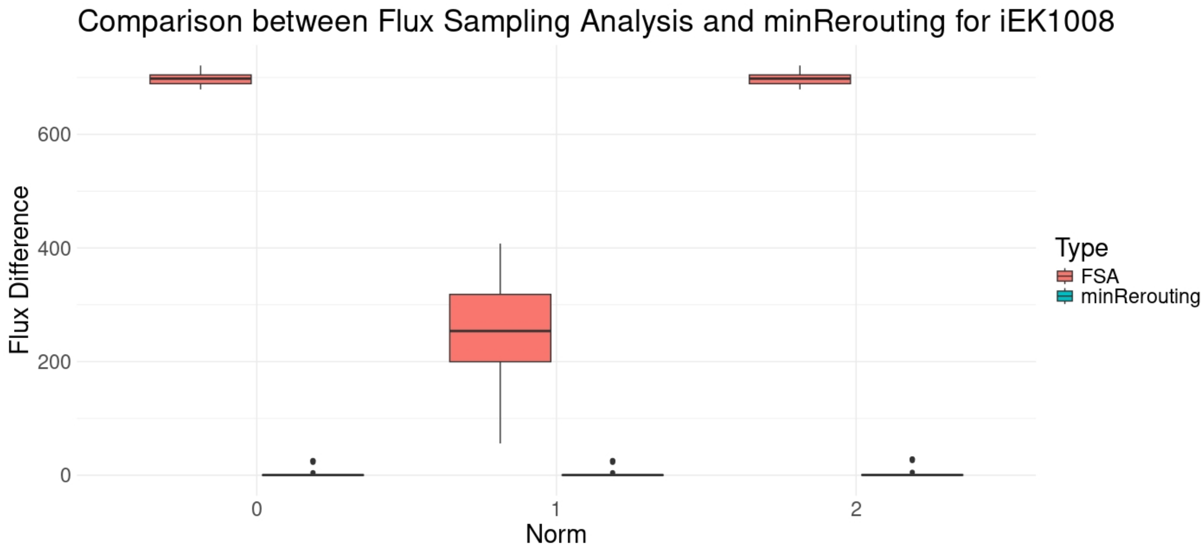

Supplementary Figure 31: **Comparison between flux sampling and minRerouting for 25 pairs of synthetic lethals for *i*EK1008.**

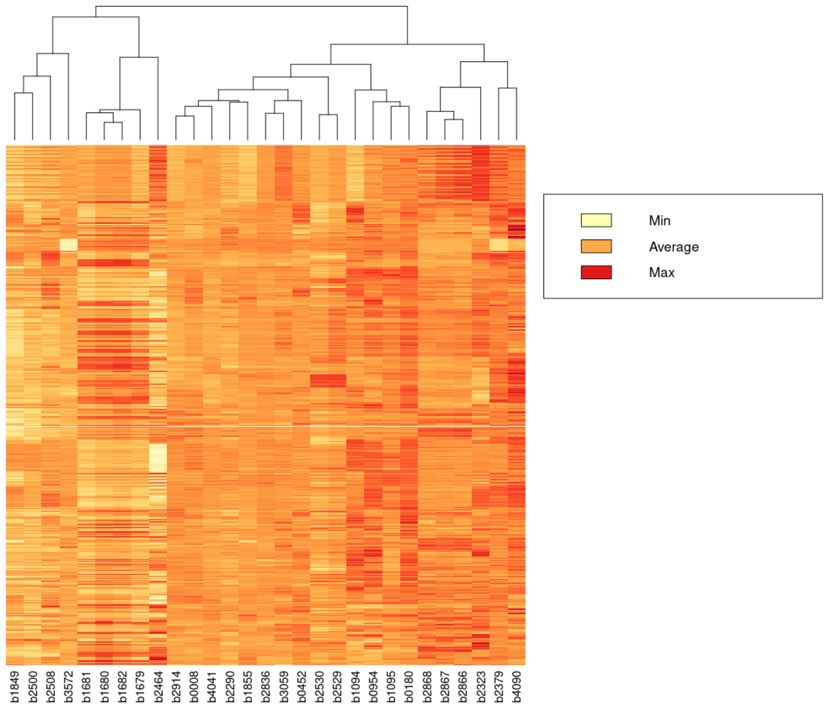

Supplementary Figure 32: **Heatmap of gene expression data from iModulonDB [2] for genes of 10 PSL synthetic lethal pairs as given in Supplementary Table 1.** The genes are clustered hierarchically. Each row represents a different condition for the gene expression study.

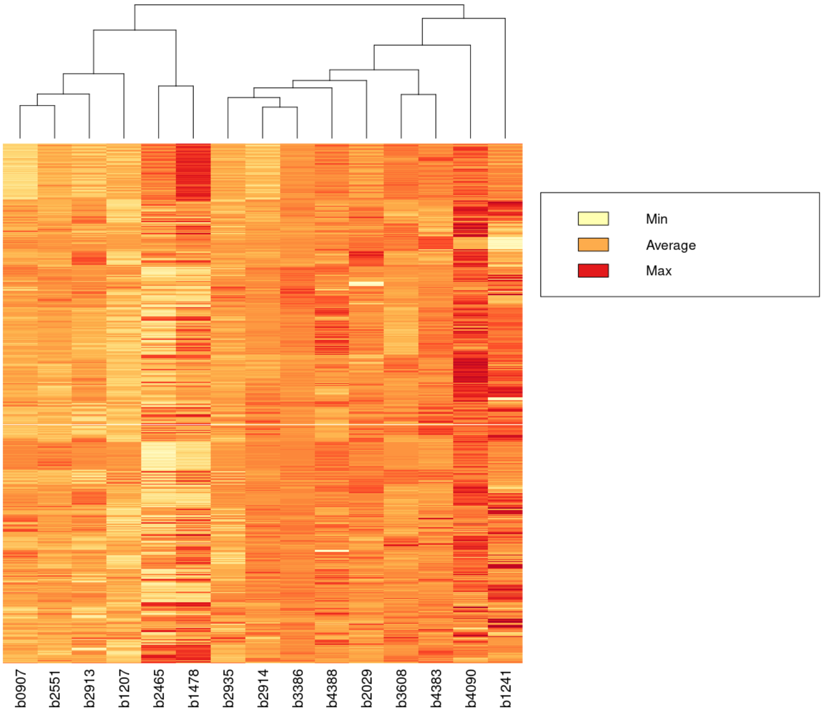

Supplementary Figure 33: **Heatmap of gene expression data from iModulonDB [2] for genes of 10 RSL synthetic lethal pairs as given in Supplementary Table 2.** The genes are clustered hierarchically. Each row represents a different condition for the gene expression study.

3 Supplementary Tables

Reaction 1	Genes for Reaction 1	Reaction 2	Genes for Reaction 2
AACPS3	b1094, b2636	3HAD160	b0954, b0180
AACPS3	b1094, b2636	3OAS160	b1095
ALATA_L	b2290, b2379	VPAMTr	b3572
FACOAE120	b0452	3HAD120	b0180, b0954
G3PAT160	b1094, b4041	APG3PAT160	b3059
GARFT	b2500	GART	b1849
I2FE2SS	b2530, b2529	S2FE2SS	b1679, b1680, b1681, b1682, b1683
IMPD	b2508	HXAND	b2866, b2867, b2868
RPI	b2914, b4090	TALA	b0008, b2464
RPI	b2914, b4090	EDTXS2	b18555

Supplementary Table 1: **Reactions and corresponding genes of 10 PSL synthetic lethal pairs.**

Reaction 1	Genes for Reaction 1	Reaction 2	Genes for Reaction 2
TKT1	b2935, b2465	TKT2	b2935, b2465
RPE	b3386	TKT2	b2935, b2465
RPI	b2914, b4090	TKT1	b2935, b2465
RPI	b2914, b4090	TKT2	b2935, b2465
PRPPS	b1207	PPM	b4383
G3PD2	b3608	ALCD19	b1478, b1241
PGCD	b2913	GHMT2r	b2551
PSERT	b0907	GHMT2r	b2551
PSP_L	b4388	GHMT2r	b2551
TKT2	b2914, b4090	GND	b2029

Supplementary Table 2: **Reactions and corresponding genes of 10 RSL synthetic lethal pairs.**

GSMM	Model_carbon	Model_exchange_1	Model_exchange_2	Model_exchange_3
Escherichia coli (e_coli_core)	13	3	3	3
Mycobacterium tuberculosis (*i*EK1008)	345	248	248	248
Helicobacter pylori 26695 (*i*IT341)	238	171	171	171
Escherichia coli (*i*ML1515)	223	112	112	112
Yersinia pestis (*i*PC815)	210	111	111	111
Shigella sonnei (*i*SSON_1240)	260	99	99	99
Klebsiella pneumoniae (*i*YL1228)	198	68	68	68
Salmonella enterica (STM_v1_0)	329	121	121	121

Supplementary Table 3: Number of single lethals obtained on using *minRerouting* after changing the environment as mentioned in the Methods.

GSMM	Model_carbon	Model_exchange_1	Model_exchange_2	Model_exchange_3
Escherichia coli (e_coli_core)	79	24	24	24
Mycobacterium tuberculosis (*i*EK1008)	156	170	170	170
Helicobacter pylori 26695 (*i*IT341)	102	105	105	105
Escherichia coli (*i*ML1515)	274	223	223	223
Yersinia pestis (*i*PC815)	186	189	189	189
Shigella sonnei (*i*SSON_1240)	260	221	221	221
Klebsiella pneumoniae (*i*YL1228)	145	145	145	145
Salmonella enterica (STM_v1_0)	157	237	237	237

Supplementary Table 4: Number of double lethals obtained on using *minRerouting* after changing the environment as mentioned in the Methods.

References

[1] Francesco Alessandro Massucci, Francesc Sagués, and M. Angeles Serrano. "Metabolic plasticity in synthetic lethal mutants: Viability at higher cost". In: *PLOS Computational Biology* 14.1 (Jan. 2018), pp. 1–20. DOI: 10.1371/journal.pcbi.1005949. URL: https://doi.org/10.1371/journal.pcbi.1005949.

[2] Kevin Rychel et al. "iModulonDB: a knowledgebase of microbial transcriptional regulation derived from machine learning". In: *Nucleic Acids Research* 49.D1 (Oct. 2020), pp. D112–D120. ISSN: 0305-1048. DOI: 10.1093/nar/gkaa810. eprint: https://academic.oup.com/nar/article-pdf/49/D1/D112/35364090/gkaa810.pdf. URL: https://doi.org/10.1093/nar/gkaa810.
